# The revealing of a novel double bond reductase related to perilla ketone biosynthesis in *Perilla frutescens*

**DOI:** 10.1186/s12870-023-04345-1

**Published:** 2023-06-30

**Authors:** Peina Zhou, Yongfang Shao, Zheng Jiang, Jingjie Dang, Cheng Qu, Qinan Wu

**Affiliations:** 1 College of Pharmacy, Nanjing University of Chinese Medicine, Nanjing, 210023 China; 2 Collaborative Innovation Center of Chinese Medicinal Resources Industrialization, Nanjing, 210023 China; 3 National and Local Collaborative Engineering Center of Chinese Medicinal Resources Industrialization and Formulae Innovative Medicine, Nanjing, 210023 China

**Keywords:** *Perilla frutescens*, Transcriptome, Double bond reductase, Enzyme assays, Perilla ketone biosynthesis

## Abstract

**Background:**

*Perilla frutescens* is widely used as both a medicine and a food worldwide. Its volatile oils are its active ingredients, and, based on the different volatile constituents, *P. frutescens* can be divided into several chemotypes, with perilla ketone (PK) being the most common. However, the key genes involved in PK biosynthesis have not yet been identified.

**Results:**

In this study, metabolite constituents and transcriptomic data were compared in leaves of different levels. The variation in PK levels was the opposite of that of isoegoma ketone and egoma ketone in leaves at different levels. Based on transcriptome data, eight candidate genes were identified and successfully expressed in a prokaryotic system. Sequence analysis revealed them to be double bond reductases (PfDBRs), which are members of the NADPH-dependent, medium-chain dehydrogenase/reductase (MDR) superfamily. They catalyze the conversion of isoegoma ketone and egoma ketone into PK in in vitro enzymatic assays. PfDBRs also showed activity on pulegone, 3-nonen-2-one, and 4-hydroxybenzalacetone. In addition, several genes and transcription factors were predicted to be associated with monoterpenoid biosynthesis, and their expression profiles were positively correlated with variations in PK abundance, suggesting their potential functions in PK biosynthesis.

**Conclusions:**

The eight candidate genes encoding a novel double bond reductase related to perilla ketone biosynthesis were identified in *P. frutescens*, which carries similar sequences and molecular features as the *MpPR* and *NtPR* from *Nepeta tenuifolia* and *Mentha piperita*, respectively. These findings not only reveal the pivotal roles of PfDBR in exploring and interpreting PK biological pathway but also contribute to facilitating future studies on this DBR protein family.

**Supplementary Information:**

The online version contains supplementary material available at 10.1186/s12870-023-04345-1.

## Background

*Perilla frutescens* (L.) is an annual herb belonging to the Lamiaceae family. It is widely cultivated in many countries, such as China, Korea, Japan, and the Himalayan regions of India and Nepal [1]. Owing to its characteristic odor, *P. frutescens* has been used as a spice, a fragrance, and as a vegetable [2]. It has also been planted as an ornamental plant in gardens for its morphological variability and attractive appearance or produced for food coloring [3]. In addition, *P. frutescens* has been used as a herb medicine for the prevention and treatment of various ailments owing to its cardiovascular effects, anti-inflammatory and rheumatoid arthritis actions, and antidepressant actions [4]. In the Chinese Pharmacopoeia (2020 version), the dried leaves, stems, and seeds of *P. frutescens* are recorded as traditional Chinese medicine (TCM) for asthma, influenza, cough, chronic bronchitis, and vomiting [1].

According to modern phytochemical and phytopharmacological research concerning *P. frutescens*, various active components have been isolated, such as monoterpenes, sesquiterpenoids, flavonoids, fatty acids, triterpenes, and phenolic compounds [5, 6]. Among these compounds, monoterpenes and sesquiterpenoids are usually present in volatile oils and are noted as active substances. Monoterpenes include perillaldehyde (PA), limonene, perilla ketone (PK), isoegomaketone, perillene (PL), elsholtzia ketone (EK), and piperitenone (PT); sesquiterpenes include *β*-caryophyllene, *α*-farnesene, and pinene [7, 8]. In China and other countries, volatile oils and their components are commonly used to evaluate the quality of *P. frutescens* [2].

Based on its volatile oil constituents, *P. frutescens* can be divided into several chemotypes, including the PA, PK, EK, PT, PL, transcitral (C), and phenylpropanoid (PP) types [9]. Among these types, PK is commonly distributed in China and other countries, with isoegoma ketone and PK being the main compounds [10, 11]. Isoegoma ketone and PK are both furanoid monoterpenes, with most being generated by the 2-C-methyl-D-erythritol-4-phosphate (MEP) pathway, and small number by the mevalonate (MVA) pathway [12]. Geranyl diphosphate (GPP) is the central precursor of all regular monoterpenoids, which are catalyzed by isopentenyl diphosphate (IPP) and dimethylallyl diphosphate (DMAPP) by geranyl diphosphate synthase (GPPS) [13]. GPP is then converted into geraniol by geraniol synthase (GES), and geraniol is transformed into geranial by geraniol dehydrogenase (GeDH) [14]. Geranial can undergo several transformations, leading to the formation of PK. Perillene and egoma ketone are intermediates in these transformations [15, 16]. However, the PK biosynthetic pathway in *P. frutescens* remains unclear. Hence, the identification of key genes related to PK biosynthesis may contribute to the genetic improvement of *P. frutescens.*

In this study, the volatile compounds of *P. frutescens* were characterized, mainly focusing on variations in PK, isoegoma ketone, and egoma ketone in different levels of leaves (Leaf 1 to Leaf 9 named as L1 to L9). Transcriptome analysis in different levels of *P. frutescens* leaves (L4, L6, L8 and L9) were performed to identify putative genes and transcription factors involved in PK biosynthesis and regulation. Expression profiles of candidate genes related to terpenoids biosynthesis were analyzed at the transcriptional level and investigated using real-time quantitative PCR (RT-qPCR). Eight candidate genes encoding PfDBR were cloned and functionally characterized through prokaryotic expression and in vitro enzyme-catalyzed reactions, suggesting that these proteins reduced isoegoma ketone and egoma ketone to PK. The ability of eight proteins to catalyze different substrates revealed the potential steric hindrance of different substrates may contribute to substrate selectivity of PfDBRs. These results not only provide a detailed understanding of PK biosynthesis and regulation in *P. frutescens* but also lay the foundation for exploring DBR function in other plants.

## Results

### Characterization of volatile compounds

**Fig. 1:**
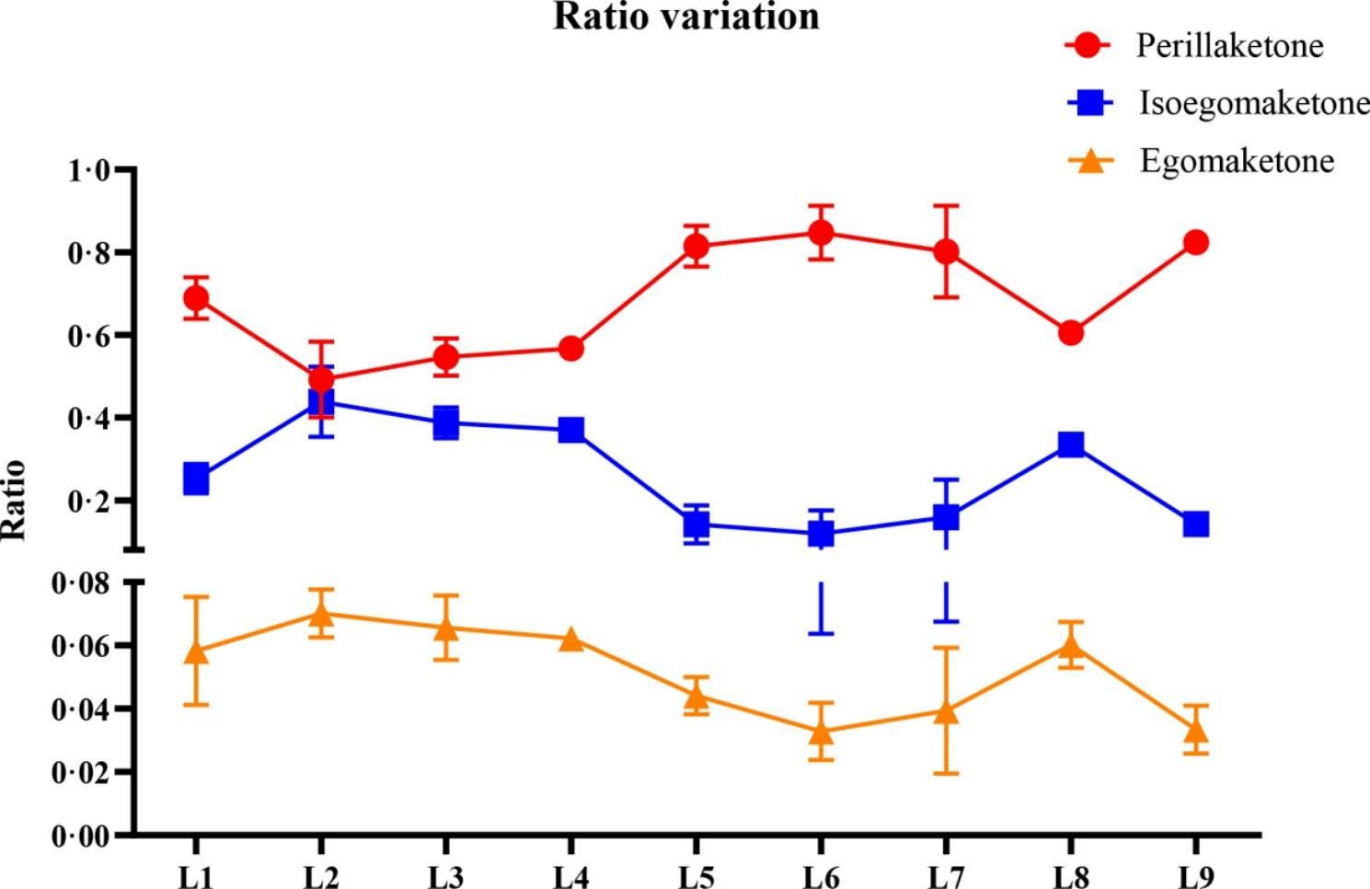
Ratio variation of perilla ketone, isoegoma ketone, and egoma ketone among leaves of different levels

In this study, GC-MS was performed to detect volatile compounds in the leaves of *P. frutescens* at different levels (Fig. S1), and analyzed the dynamic changes in the accumulation of metabolites in different leaves. A total of eight volatile compounds were identified in *P. frutescens* leaves by standards and NIST library (Table 1). The relative contents of PK, isoegoma ketone, egomak etone, *β*-caryophyllene, and (Z, E)-*α*-farnesene accounted for more than 98%. PK, isoegoma ketone and egoma ketone accounted for more than 80% of their average from L1 to L9 (Table 1), showing the *P. frutescens* in this study was PK type. To explore the variation trends in PK, isoegoma ketone, and egoma ketone, we regarded the peak areas of these three compounds as 1 to analyze the changes in their proportions (Table S1). PK always accounted for the highest proportion, followed by isoegoma ketone, and egoma ketone appeared to be the lowest, and the proportion was one order of magnitude lower than that of PK and isoegoma ketone. Isoegoma ketone and egomake tone exhibited similar variation trends, which were opposite to that of PK (Fig. 1).

**Table 1:** Volatile compounds of *P. frutescens*

ID	Compounds	Retention time (RT)	CAS	Foluma	Type	Relative content (%)								
						L1	L2	L3	L4	L5	L6	L7	L8	L9
1	Linalool	9.731	78-70-6	C_10_H_18_O	Monoterpenoid	0.78	1.09	0.25	0.28	0.66	1.22	0.47	0.25	0.26
2	Perilla ketone*	14.011	553-84-4	C_10_H_14_O_2_	Monoterpenoid	65.65	37.80	52.34	48.11	60.07	64.57	56.10	52.36	65.40
3	Egoma ketone*	15.491	59204-74-9	C_10_H_12_O_2_	Monoterpenoid	3.88	6.48	4.76	5.15	3.50	1.74	5.16	5.58	3.15
4	Isoegoma ketone*	15.697	34348-59-9	C_10_H_12_O_3_	Monoterpenoid	18.72	40.14	31.28	29.29	14.74	6.05	21.46	27.75	13.80
5	*β*-Caryophyllene*	19.083	87-44-5	C_15_H_24_	Sesquiterpenoid	5.67	4.98	5.18	7.58	8.75	11.10	6.76	5.80	7.78
6	*α*-Caryophyllene	19.975	6753-98-6	C_15_H_24_	Sesquiterpenoid	NA	0.03	0.12	0.19	0.42	0.33	0.25	0.38	0.53
7	Germacrene D	20.692	23986-74-5	C_15_H_24_	Sesquiterpenoid	0.33	NA	0.33	0.42	0.99	1.00	0.72	0.66	1.06
8	*(Z, E)-α*-farnesene	20.999	26560-14-5	C_15_H_25_	Sesquiterpenoid	4.97	9.48	5.75	8.97	10.87	13.99	9.08	7.22	8.03

* represented compounds were confirmed with standards

### Transcriptome assembly and annotation

To explore PK biosynthesis, we performed transcriptome analysis of four leaf levels (L4, L6, L8, and L9) where the relative contents of PK, egoma ketone, and isoegoma ketone varied greatly. Twelve libraries were constructed and sequenced from samples L4, L6, L8, and L9. A total of 75.44 Gb of clean data were obtained from these libraries. After removing adapters and reads with low quality, the percentage of clean reads in all 12 transcriptomes exceeded 99%. The average values of Q20 and Q30 were more than 97% and 92%, respectively (Table S2). More than 94% of the reads of these libraries mapped to the genome. The above results suggested the good quality of the 12 transcriptome libraries. Based on the *P. frutescens* genome and StringTie algorithm, there were 49,173 genes, including 10,232 novel genes and 38,941 mapped genes. 35,420 genes were annotated into GO, including 16 groups for molecular functions (MF), 26 groups for biological processes (BP), and 23 groups for Cellular Compent (CC) (Fig. S2A). Most genes were involved in cellular processes, metabolic processes, and binding terms. In KEGG analysis, it was 10,118 genes annotated which were mapped into 139 pathways, with 108 pathways into metabolism, 21 pathways into genetic information processing, 4 pathways into cellular processes, 4 pathways into environmental information processing, and 2 pathways into organismal systems (Fig. S2B). Pathways such as plant hormone signal transduction, phenylpropanoid biosynthesis, and biosynthesis related to terpenoids, fatty acids, flavonoids, and amino acids were enriched in many genes, suggesting that the genes involved in these pathways may be worthy of attention. Based on the criteria of FDR < 0.05, and |log2FC| ≥ 1, a total of 17,516 DEGs were obtained by pairwise comparison of four groups (L4, L6, L8, and L9). There were 14,109 DEGs in L9 vs. L4 (5,092 upregulated, 9,017 downregulated), 11,070 DEGs in L8 vs. L4 (4,586 upregulated, 6,484 downregulated), 11,015 DEGs in L9 vs. L6 (3,357 upregulated, 7,658 downregulated), 6,030 DEGs in L8 vs. L6 (1,941 upregulated, 6,030 downregulated), 5,149 DEGs in L9 vs. L8 (1,327 upregulated, 3,822 downregulated), and 3,542 DEGs in L6 vs. L4 (1,609 upregulated, 1,933 downregulated) (Fig. S3). These DEGs were assigned to 137 KEGG pathways, and phenylpropanoid biosynthesis, flavonoid biosynthesis, fatty acid biosynthesis, and cutin, suberin, and wax biosynthesis were all enriched in the above comparisons (Fig. S4).

### Identification of transcription factors

Transcription factors (TF) often regulate target metabolic pathways globally and dynamically; therefore, the Plant Transcription Factor Database (Plant TFDB) was used to search for protein domains to identify genes encoding TFs in *P. frutescens*. A total of 2,487 genes were annotated as TFs. Among these genes, 791 were DEGs that may regulate metabolic pathways in *P. frutescens*. Most TFs were enriched in the MYB (89 DEGs), AP2/ERF (85 DEGs), and bHLH (73 DEGs) families. These TF families are believed to be involved in the synthesis of terpenoids and other metabolites [17]. Most MYB and bHLH TFs were highly expressed in L9, while AP2-ERF TFs were highly expressed in L4 and L9 (Fig. S5, Table S3). AP2-ERF play very important roles in plant growth, plant stress responses, and secondary metabolism, such as artemisinin, nicotine, and anthocyanin [18, 19], and thus the DEGs annotated as the AP2-ERF family may be expressed in different stages of leaves. MYB and bHLH were among the largest groups of TFs, and MYB often interacts with bHLH to generate MYB-bHLH complexes [20]. Hence, the DEGs noted as *MYB* and *bHLH* had similar expression patterns. These complexes are known to regulate plant development and metabolic pathways [21]. They may affect the fate of glandular trichomes (GTs) during leaf development, and the volatile oil of *P. frutescens* is mainly accumulated in its peltate glandular trichomes (PGTs) [22].

### Analysis of monoterpenoid and other terpenoids biosynthesis

**Fig. 2:**
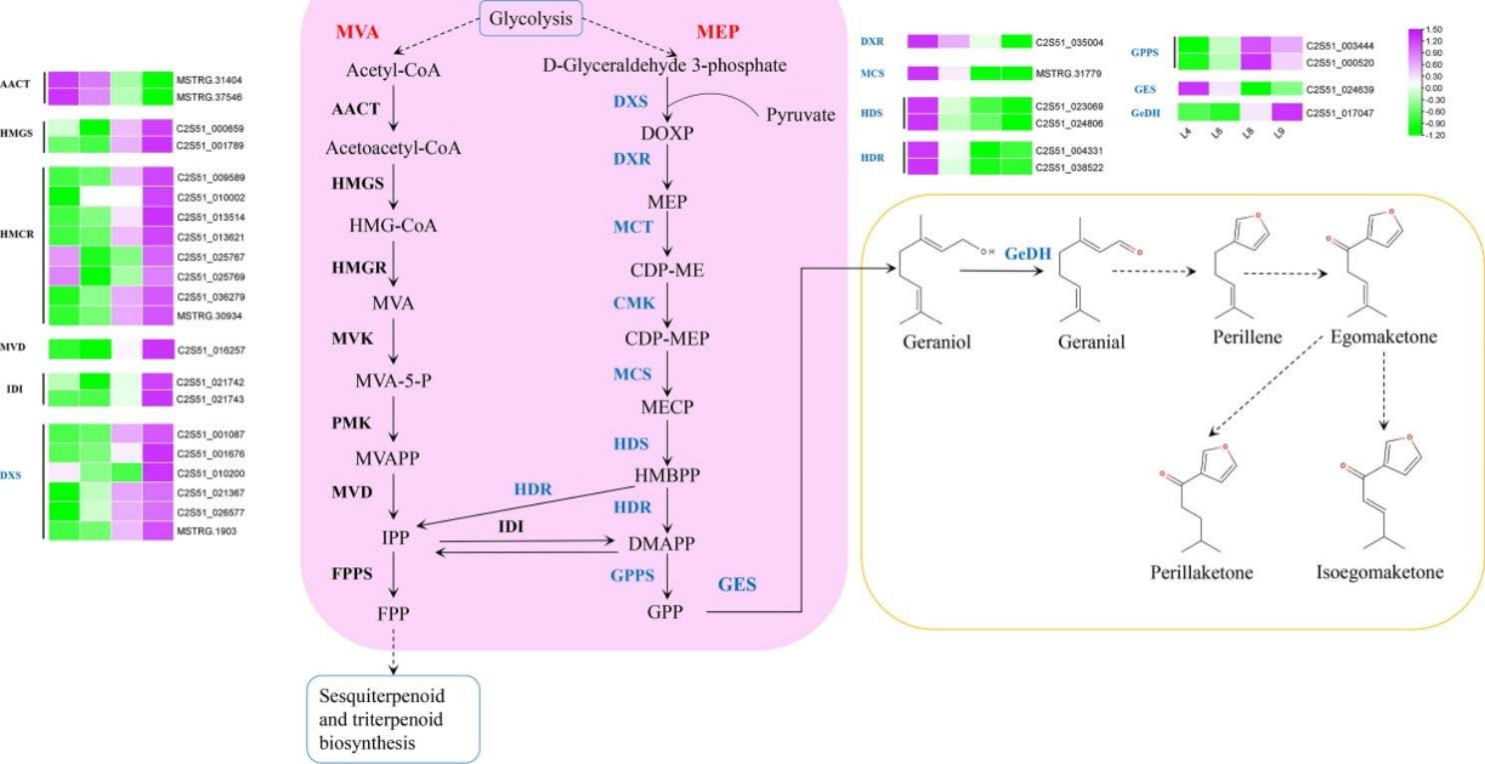
Biosynthesis of terpenoids and expression of related DEGs across four levels of *P. frutescens* leaves. Highly expressed genes are indicated in purple, whereas lowly expressed genes are in green. From left to right, the four levels were L4, L6, L8, and L9. Genes in blue are involved in the MEP and monoterpenoid biosynthesis pathways, and genes in black are involved in the MVA pathway. Dashed arrows in the yellow boxes indicate unknown biosynthetic pathways of perilla ketone. Abbreviations: DOXP, 1-Deoxy-D-xylulose 5-phosphate; MEP, 2-C-Methyl-D-erythritol 4-phosphate; CDP-ME, 4-(Cytidine 5’-diphospho)-2-C-methyl-D-erythritol; CDP-MEP, 2-Phospho-4-(cytidine 5’-diphospho)-2-C-methyl-D-erythritol; MECP, 2-C-Methyl-D-erythritol 2,4-cyclodiphosphate; HMBPP, 1-Hydroxy-2-methyl-2-butenyl 4-diphosphate; IPP, isopentenyl diphosphate; DMAPP, dimethylallyl diphosphate; AACT, acetyl-CoA acetyltransferase; HMGS, hydroxymethylglutaryl-CoA; HMGCR, hydroxymethylglutaryl-CoA reductase synthase; MVK, mevalonate kinase; PMK, phosphomevalonate kinase; MVD, mevalonate diphosphate decarboxylase; MVA, Mevalonic acid; MVA-5-P, Mevalonic acid 5-phosphate; MVAPP, 5-Diphosphomevalonic acid; FPP, Farnesyl diphosphate. The biosynthesis was redrawn based on map 00900 and 00902 (https://www.genome.jp/pathway/map00900, https://www.genome.jp/entry/map00902)

To explore the regulatory mechanisms underlying the accumulation patterns of different terpenoids in leaves at different levels, the expression profiles of genes involved in terpenoid biosynthesis were analyzed. Based on KEGG pathway analysis, 32 DEGs were identified in terpenoid backbone biosynthesis, 19 in monoterpenoid biosynthesis, 15 in diterpenoid biosynthesis, and 29 in sesquiterpenoid and triterpenoid biosynthesis. Most genes in the MVA pathway were highly expressed in L9, whereas genes related to the MEP pathway were upregulated in L4 (Table S4). PK was generated from geranial which were transformed from GPP by GPPS. Geranial was produced from geraniol by geraniol dehydrogenase (GeDH), which was upregulated in L9 (Fig. 2). Most DEGs involved in monoterpenoid, sesquiterpenoid, and triterpenoid biosynthesis were highly expressed in L9, and DEGs in diterpenoid biosynthesis were highly expressed in L4 (Fig. S6).

**Fig. 3:**
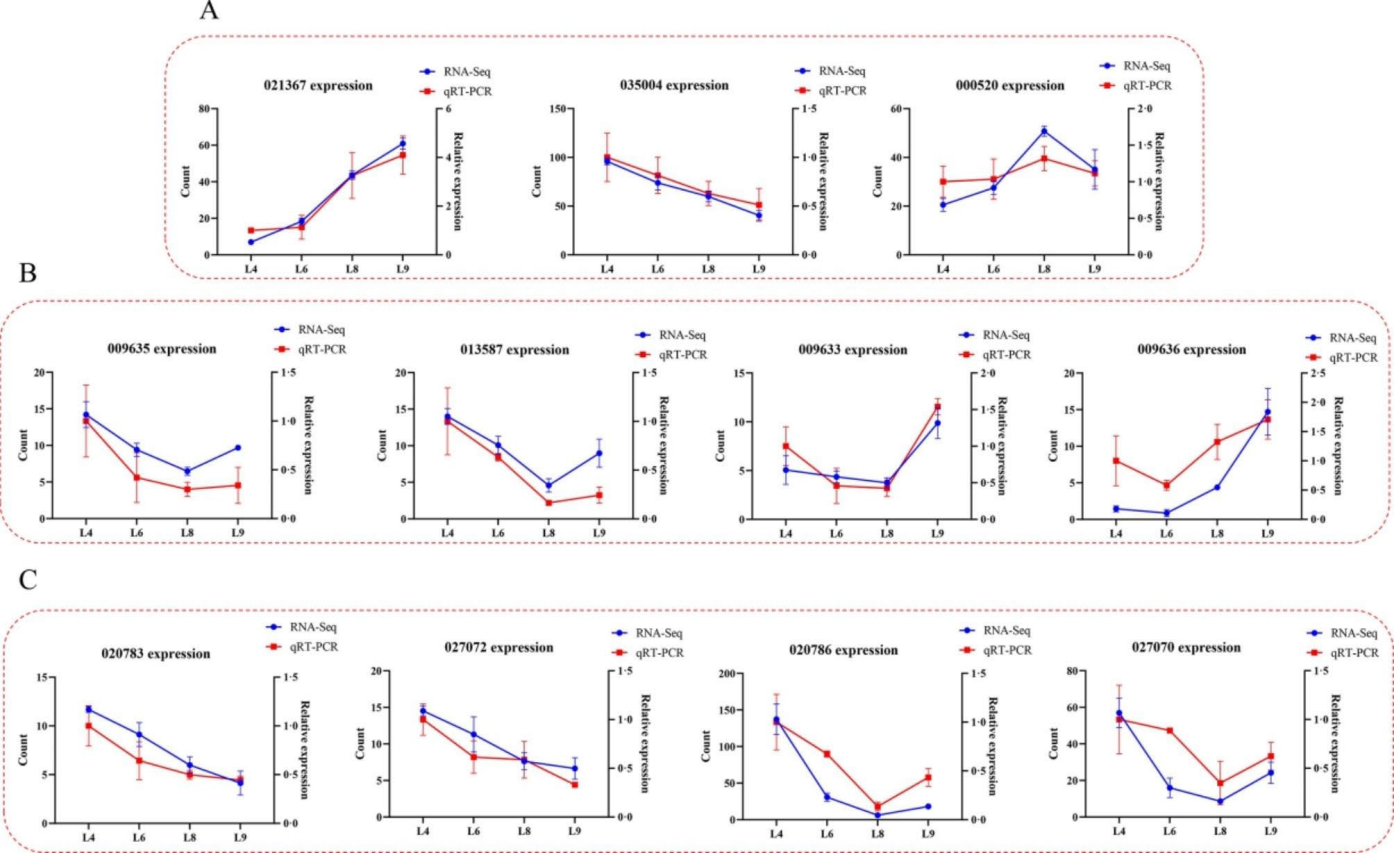
Gene expression in RNA-Seq and RT-qPCR. **(A)** Expression of genes annotated as *DXR*, *DXS*, and *GPPS*. **(B)** Expression of homologous genes of *NtPR*. **(C)** Expression of homologous genes of *MpPR*. Three independent biological replicates were used

PK is the major component of *P. frutescens*, which is generated from geraniol, the downstream compound of GPP. GPP is typically derived from the MEP pathway. The genes for encoding 1-deoxy-D-xylulose-5-phosphate synthase (DXS), GPPS, and GeDH were highly expressed in the youngest L9 leaves. In contrast, genes encoding 1-deoxy-D-xylulose-5-phosphate reductoisomerase (DXR), 2-C-methyl-D-erythritol 2,4-cyclodiphosphate synthase (MCS), (E)-4-hydroxy-3-methylbut-2-enyl-diphosphate synthase (HDS), 4-hydroxy-3-methylbut-2-en-1-yl diphosphate reductase (HDR), and geranyl diphosphate diphosphatase (GES) were upregulated in matured L4 leaves. The transcripts of genes encoding 2-C-Methyl-D-eryth-ritol 4-phosphate cytidylyltransferase (MCT) and 4-diphosphocytidyl-2-C-methyl-D-erythritol kinase (CMK) were not found in the DEGs. Transcripts C2S51_035004, C2S51_021367, and C2S51_000520 were annotated as *DXR*, *DXS*, and *GPPS*, respectively, and their expression in RNA-Seq was validated by RT-qPCR (Fig. 3).

To integrate the related gene expression with three main compound variations, Pearson’s correlation analysis was established between the ratio of three main compounds (PK, isoegoma ketone, and egoma ketone) and related gene expression patterns (MVA, MEP pathway genes, *GES*, and *GeDH*) at different leaf levels (Table S5). From the results of the above-mentioned correlation analysis, genes C2S51_001676_DXS, C2S51_010002_HMGR, and C2S51_013514_HMGR were negatively correlated with isoegoma ketone and egoma ketone and positively correlated with PK (*p* > 0.6). Thus, these genes may play essential roles in PK biosynthesis.

### Identification of DBR

**Fig. 4:**
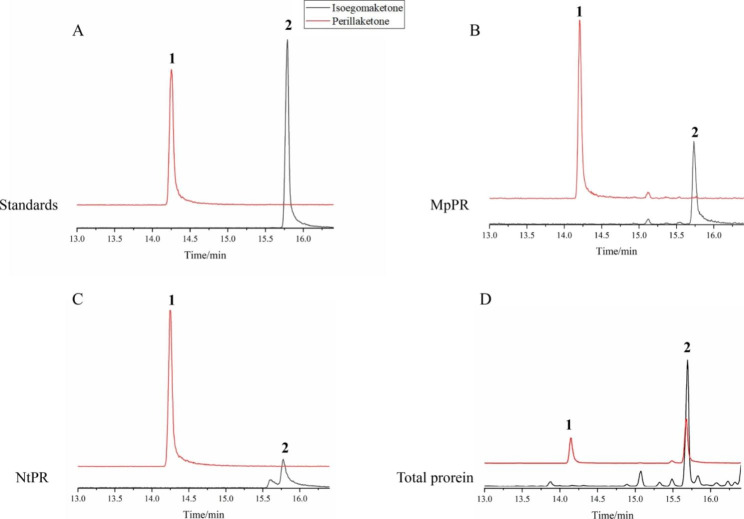
Functional characterization of proteins from *P. frutescens* leaves. (A) perilla ketone (peak 1, RT 14.25 min) and isoegoma ketone (peak 2, RT 15.86 min) standards. (B) Reaction of isoegoma ketone to perilla ketone catalyzed by proteins from *P. frutescens* leaves. (C-D) Reaction of isoegoma ketone to perilla ketone catalyzed by MpPR and NtPR. The black line in B-D indicated the experimental group, and the red line indicated negative control. The protein in negative control was boiled and other ingredients were same as experimental group

PK, isoegoma ketone, and egoma ketone have not yet been investigated for their biosynthetic potential. Based on their chemical structures, we speculated that a DBR existing in *P. frutescens* plants could utilize egoma ketone or isoegoma ketone as substrates to synthesize PK, but such a reductase has not yet been reported. Thus, we extracted proteins from *P. frutescens* leaves to conduct in vitro enzymatic assays, whereby the protein extract was mixed with isoegoma ketone. The products were analyzed by GC-MS, and the compounds were confirmed by comparison with known standards (Fig. 4A). Isoegoma ketone was converted into PK as compared with the negative control (Fig. 4B), showing that there was a reductase in *P. frutescens* reducing the C7-C8 double bond of isoegoma ketone to PK. In *Mentha piperita* and *Nepeta tenuifolia*, isopiperitenone is converted into pulegone by isopiperitenone isomerase (IPI), which is then converted to menthone by pulegone reductases (PRs). This reaction is similar to the conversion of compounds from egoma ketone to isoegoma ketone, followed by PK. Thus, we expressed MpPR and NtPR in *E. coli* BL21 (DE3) and conducted enzymatic assays in vitro, in which isoegoma ketone was added to the reaction and the products detected and analyzed using GC-MS. Based on our previous report and standards, the results clearly showed that isoegoma ketone was completely converted to PK in the MpPR and NtPR reactions, confirming that MpPR and NtPR can reduce the double bond of isoegoma ketone (Fig. 4C-D).

### Isolation and functional analysis of DBRs in *P. frutescens*

Both NtPR and MpPR can react with isoegoma ketone to form PK, which has the same function as the total protein extract of *P. frutescens*. We speculated that the DBRs of *P. frutescens* may have similar amino acid sequences to NtPR and MpPR. These proteins all belong to the NADPH-dependent, medium-chain dehydrogenase/reductase (MDR) superfamily. First, candidate PfDBRs were identified from the RNA-Seq data using NtPR and MpPR sequences as queries. DEGs were selected among these genes, and C2S51_020783, C2S51_027072, C2S51_020786, and C2S51_027070 were similar to MpPR, and C2S51_009635, C2S51_013587, C2S51_009633, and C2S51_009636 were similar to NtPR. Multiple sequence alignment indicated that the above eight proteins shared a high sequence identity (80.76%) with MpPR and NtPR, and that 10 proteins belonged to the MDR superfamily from plants (Fig. S7). A total of 43 DBRs, including MDR superfamily members in NCBI and the eight selected genes above, were chosen for maximum-likelihood (ML) phylogenetic analysis. As expected, the phylogenetic tree revealed that C2S51_020783, C2S51_027072, C2S51_020786, and C2S51_027070 were closely related to MpPR and McPR, and C2S51_009635, C2S51_013587, C2S51_009633, and C2S51_009636 were closely related to NtPR (Fig. S8). RT-qPCR of the eight genes was also performed, and gene expression patterns were consistent with RNA-Seq results (Fig. 3).

**Fig. 5:**
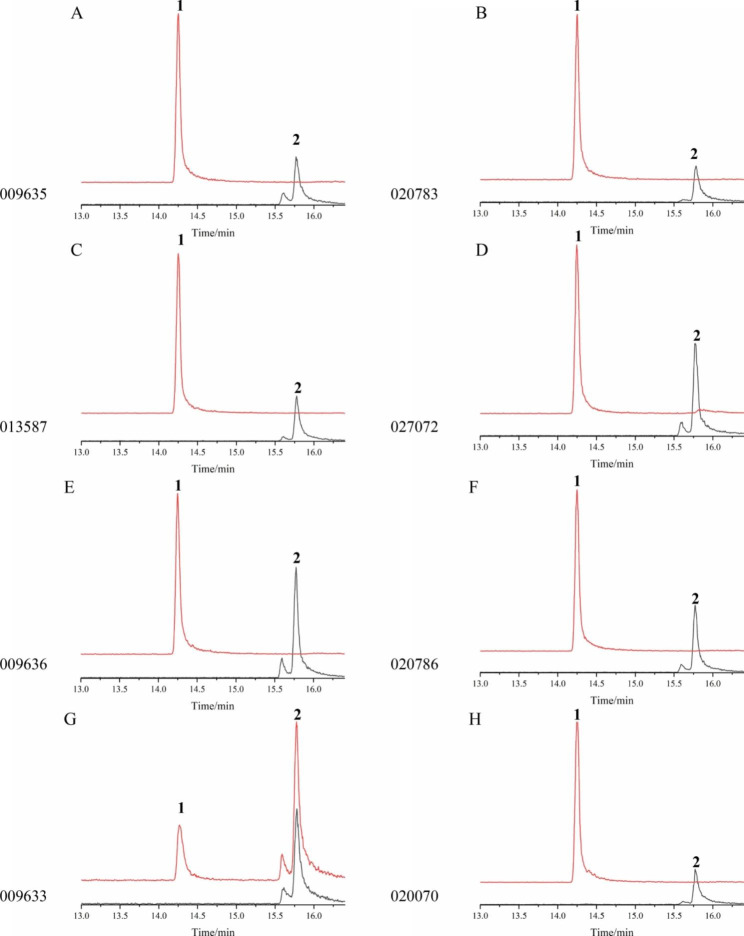
Functional characterization of eight proteins. (A-H) Reaction of isoegoma ketone to perilla ketone catalyzed by C2S51_009635 (A), C2S51_020783 (B), C2S51_013587 (C), C2S51_027072 (D), C2S51_009636 (E), C2S51_020786 (F), C2S51_009633 (G), and C2S51_027070 (H). Peak 1 was perilla ketone with RT 14.25 min, and peak 2 was isoegoma ketone with RT 15.86 min. The red line indicated the experimental group, and the black line indicated negative control. The protein in negative control was boiled and other ingredients were same as experimental group

Third, the eight homologous coding regions were cloned into vector pET28a to express their encoded proteins, which were then subjected to in vitro enzymatic assays. Protein extracts were mixed with isoegoma ketone and analyzed by GC-MS. The results showed that all eight proteins converted isoegoma ketone into PK, suggesting that they may act as PfDBRs (Fig. 5).

To investigate the substrate specificity of these eight proteins, we added substrates with similar double bonds, such as pulegone, 3-nonen-2-one, 4-hydroxybenzalacetone, artemisinic acid, and artemisitene. GC-MS was used to analyze the reaction products of pulegone and 3-nonen-2-one, revealing that all eight proteins converted pulegone (RT 14.01 min) into menthone (RT 11.37 min) and isomenthone (RT 11.63 min), and converted 3-nonen-2-one (RT 10.83 min) into 2-nonanone (RT 9.60 min) (Fig. S9-S10). 4-Hydroxybenzalacetone with RT 17.78 min was converted into raspberry ketone with RT 16.90 min by the eight proteins based on HPLC analysis (Fig. S11). However, HPLC results for proteins mixed with artemisinic acid (RT 13.25 min) and artemisitene (RT 5.38 min) revealed that these two substrates were unchanged (Fig. S12-SS13). The dihydroartemisinic acid (RT 11.71 min) and artemisinin (RT 6.25 min), the reduction products of artemisinic acid and artemisitene, respectively, were not observed in the HPLC peaks. The results of in vitro enzymatic assays showed that these reductases are selective for substrate structures.

## Discussion

*P. frutescens* is a spicy vegetable and medicinal herb that is widely cultivated in many countries, especially in Korea, China, Japan and other Asian regions. Its leaves can be green, purple, with a green adaxial leaf and a purple abaxial leaf featuring several morphological characteristics [23]. There are seven chemotypes of *P. frutescens* based on different oil constituents, and the PK chemotype is commonly used in China, usually with green adaxial leaves and purple abaxial leaves, enriched in isoegoma ketone and PK [22]. However, the biosynthetic pathways involving isoegoma ketone and PK remain to be fully elucidated, although genes encoding proteins that catalyze GPP to geranial have been characterized [16]. In this study, we compared metabolite constituents and transcriptomic data among L4, L6, L8, and L9 leaves of PK type *P. frutescens*. Based on RNA-Seq and bioinformatic analyses, a novel double bond reductase that convert isoegoma ketone into PK was identified and its functions were analyzed using in vitro enzymatic assays.

The compound egoma ketone was one of the product in PK biosynthesis, and detected in *P. frutescens* leaves. It has same molecular formula with isoegoma ketone, and based on chemical structure comparisons, the difference between isoegoma ketone and egoma ketone is only the position of the double bond (Fig. 1). We speculated that may have isomerization of the double bonds between isoegoma ketone and egoma ketone which is similar to the conversion of isopulegone and pulegone in *N. tenuifolia* and *M. piperita.* Subsequently, the C = C double bond conjugated with the carbonyl group of pulegone is reduced to produce menthone or isomenthone. The structural transformation of isoegoma ketone to PK also involves the reduction of the C = C double bond conjugated with the carbonyl group. Therefore, PfDBRs may have a reduction function similar to that of NtPR and MpPR. Eight homologous genes of *NtPR* and *MpPR* were selected for multiple sequence alignment and shared 80.76% sequence similarity; these were termed PfDBRs. Artemisinic aldehyde Δ11(13) reductase (AaDBR2) and double bond reductase (AaDBR3) in *Artemisia annua* both reduce double bond structures conjugated with carbonyl. AaDBR2 acts on artemisinic aldehyde to produce dihydroartemisinic aldehyde and reacts with 2-cyclo-hexen-1-one, carvone, and 2*E*-nonenal. No AaDBR2 activity is detected with arteannuin B, artemisinic acid, artemisinic alcohol, artemisitene, coniferyl aldehyde, pinene, pulegone, or sabinone [24]. In contrast, AaDBR3 acted on 3-nonen-2-one to generate 2-nonanone, and could not react with arteannuin B, artemisinic acid, artemisinic alcohol, or artemisitene [25]. The sequences of the above proteins and other DBRs were compared in a ML tree, revealing that AaDBR2 did not cluster with any sequences, and NtPR, MpPR, AaDBR3, and PfDBRs were in one cluster (Fig. S9). Multiple sequence alignment of NtPR, MpPR, AaDBR3, and PfDBRs showed high similarity (79%) among these sequences, which contained a well-conserved binding site (AASGAV) of NAD(P) + and NAD(P)H (Fig. S7). This structure binds to the phosphate groups of NAD(P) + and NAD(P)H. In addition, there was a highly conserved sequence of CGXXSXY, which is conserved in the MDR superfamily [25]. Based on their sequence analyses, the functions of NtPR, MpPR, AaDBR3, and PfDBRs may differ from those of AaDBR2, even though they all belong to the MDR superfamily [26].

To explore the function of PfDBRs in PK biosynthesis, eight genes were expressed in *E. coli* to obtain their encoded proteins. In vitro enzymatic assays were performed using isoegoma ketone. GC-MS results showed that the new peak from experimental samples had the same retention time as PK standard, with excellent catalytic activity (Fig. 5). These results confirmed that eight PfDBRs acted as DBRs, reducing isoegoma ketone to PK. At the same time, NtPR and MpPR were also mixed with isoegoma ketone, which also had a PK peak in the GC-MS analysis (Fig. S14). To further explore the substrate preference of PfDBRs, these eight proteins were mixed with pulegone, 3-nonen-2-one, 4-hydroxybenzalacetone, artemisinic acid, and artemisitene. The results showed that pulegone, 3-nonen-2-one, and 4-hydroxybenzalacetone were converted into menthone and isomenthone, 2-nonanone, and raspberry ketone, respectively, and that C = C double bond structure conjugated with carbonyl was reduced (Fig. S9-S11). The artemisinic acid and artemisitene were not reduced, although they had a double bond structure (Fig. S12-SS13). NtPR and MpPR each both had the same catalytic activity toward pulegone, 3-nonen-2-one, 4-hydroxybenzalacetone, artemisinic acid, and artemisitene as the eight proteins (Fig. S14-SS15). The number of carbon atoms and spatial structure of artemisinic acid and artemisitene was greater than that of pulegone, 3-nonen-2-one, and 4-hydroxybenzalacetone, which may result in the C = C double bond of the carbonyl conjugate not being well exposed exterior to the structure, blocking the reduction reaction. DBRs tend to accept small molecules as substrates, which limits the scope of their applicability [26]. The presence of a ketone or aldehyde group conjugated with C = C is likely to be fundamental for substrate activity by DBRs [27]. It has been shown that even if the above proteins like PfDBRs, NtPR, MpPR, AaDBR2 and AaDBR3 were DBRs, their catalytic substrates are different, and the catalytic mechanisms involved need to be further studied.

Biosynthesis of egoma ketone, isoegoma ketone, and PK has been conducted by cross-breeding, and the isotope-tracer method revealed that egoma ketone is the substrate of isoegoma ketone and PK, and that isoegoma ketone cannot be converted into PK [28, 29]. However, in our study, isoegoma ketone was reduced to PK by PfDBRs with good catalytic activity. In the negative control of the enzymatic assays, an egoma ketone peak was also detected, the peak on the left of isoegoma ketone peak (Fig. 5). Compared with control, both egoma ketone and isoegoma ketone were converted into PK in the experimental group, because there was only one PK peak was detected in experimental group. Thus, we speculated that both egoma ketone and isoegoma ketone might be reduced to PK by PfDBRs. However, because of the lack of an egoma ketone standard, in vitro enzymatic assays for egoma ketone could not be performed. The isoegoma ketone had C = C-conjugated double bonds, whereas egoma ketone did not have resulting in its unstable structure. In the isoegoma ketone standard, egoma ketone peaks can be detected and identified with the MS spectrum (Fig. S16). The MS spectrum of egoma ketone peak was same as that in previous studies reported [22, 30]. Thus, it is possible that isoegoma ketone and egom aketone could be converted into each other and both could be reduced to PK by PfDBRs in *P. frutescens*. According to previous researches, and our results, the PK biosynthesis from geraniol to PK may be as follows: the geranial was produced by GeDH, then had ring-closure, C6 oxidation to produce egoma ketone; egoma ketone can transform to isoegoma ketone by isomerase, and they can be reduced by PfDBR to produce PK (Fig. S17).

## Conclusion

In this study, we presented conclusive evidence for the volatile components of different levels of *P. frutescens* leaves and variations in PK, isoegoma ketone, and egoma ketone abundance. Transcriptome analysis was performed with different levels of leaves to explore a new PfDBR involved in PK biosynthesis. Eight candidate genes encoding PfDBRs were identified that reduced isoegoma ketone and egoma ketone to PK. In vitro enzymatic assays of different substrates involving MpPR, NtPR, and eight PfDBRs in this study revealed their substrate preference. These results provide insights into the complete elucidation of PK biosynthesis pathway and a valuable basis to explore DBR function in plants.

## Methods

### Plant materials

Seeds of Perilla (*P. frutescens* L.) accessions were purchased from a medicine market in Anguo City, Hebei Province, China. Seeds were planted in a nutritive soil (Miracle-Gro, Ohio, USA) in a light incubator with a day/night cycle of 16/8 h, at 25℃ and 60% relative humidity in Nanjing University of Chinese Medicine. After one month, young plants were transplanted into the greenhouse. After three months, the leaves were harvested as plant material with three replicates. The leaves were frozen in liquid nitrogen and stored at -80℃ for RNA-Seq and metabolite analysis. The plant materials were provided by Professor Qinan Wu from the College of pharmacy, Nanjing University of Chinese Medicine. The leaf 1 to leaf 9 was referred to the leaves at level 1 to 9 which were counted from the root up, which were named as L1 to L9.

### Chemical standards

D(+)-Camphor (98% purity, CAS No: 464-49-3) used as internal standard, perilla ketone (CAS No: 553-84-4), dihydroartemisinic acid (CAS No: 85031-59-0) and artemisinin (CAS No: 63968-64-9) standards were purchased from Shanghai Yuanye Biotechnology Co. LTD (Shanghai, China). The isoegoma ketone (CAS No: 34348-59-9) and raspberry ketone (CAS No: 5471-51-2) standard was purchased from Chengdu DeSiTe Biological Technology Co., Ltd (Chengdu, China). β-Caryophyllene (CAS No: 87-44-5) was purchased from Beijing Solarbio Science & Technology Co.,Ltd (Beijing, China). (+)-Pulegone (CAS No: 89-82-7) and (-)-menthone (CAS No: 14073-97-3) standards were purchased from Sigma-Aldrich (Shanghai, China). (+)-Isomenthone (CAS No: 1196-31-2), 3-nonen-2-one (CAS No: 14309-57-0), 2-nonanone (CAS No: 821-55-6) standards were purchased from Shanghai Macklin Biochemical Technology Co., Ltd (Shanghai, China). Artemisinic acid (CAS No: 80286-58-4) was purchased from Chengdu Biochem Pure Biotechnology Co., LTD (Chengdu, China). 4-hydroxybenzalacetone (CAS No: 22214-30-8) standard was purchased from Nantong Feiyu Biotechnology Co., LTD (Nantong, China).

### Extraction of volatile oils and gas chromatography-mass spectrometry (GC-MS) analysis

The leaves of *P. frutescens* were harvested to extract volatile oil. A volume of 1.8 mL of camphor internal standard (156.0 µg mL^− 1^) was added to 100 mg of leaves. The mixture was ground and treated with ultrasound three times (60 Hz, 90 s). After 12,000 *g* centrifugation for 5 min, the supernatant was collected and anhydrous sodium sulfate was added to remove water from the supernatant, passed through a 0.45 μm filter membrane, and transferred to an auto sampler vial prior to injection. The vials were stored at -80℃. All experiments were performed in triplicate.

The collected samples were analyzed using gas chromatography-mass spectrometry (GC-MS) 7890/5975 C (Agilent Technologies, Santa Clara, CA, USA) equipped with an HP-5ms fused silica column (Agilent 19,091 S-433, 30 m × 250 μm × 0.25 μm) with helium as the carrier gas (1 mL min^− 1^). The injection volume used was 1 µL. The injection temperature was 220℃, and the solvent delayed for 3 min. The programmed temperature profile was as follows: 50℃ held for 3 min, increased at a rate of 10℃ min^− 1^ to 100℃, held for 3 min, finally increased at a rate of 5℃ min^− 1^ to 200℃ and held for 3 min. The EI ion source was 70 eV electron energy, and detector temperature of the ion source was 230℃, with a mass range of 50 to 500 *m*/*z*. ChemStation software (Agilent Technologies) was used for the data acquisition and processing. Compounds were identified by standards and comparison of their mass spectra against NIST.

### RNA extension, transcriptome analysis, and RT-qPCR

Total RNA was extracted using TRIzol reagent kit (Invitrogen, Carlsbad, CA,USA) according to the manufacturer’s protocol. Then, the cDNA fragments were purified using the QiaQuick PCR extraction kit (Qiagen, Venlo, Netherlands), end-repaired, poly(A) added, and ligated to Illumina sequencing adapters. Sequencing was performed using an Illumina HiSeq2500 by Gene Denovo Biotechnology. (Guangzhou, China). Approximately 4.8 Gb of clean reads was generated from each sample. Thus, to obtain high-quality clean reads, they were further filtered using fastp (version 0.18.0). An index of the reference genome was built and paired-end clean reads were mapped to the reference genome using HISA T2. 2.4 with “-rna-strandness RF” and other parameters set as a default. Gene expression of fragments per kilobase of transcript per million mapped reads (FPKM) was calculated using StringTie v1.3.1. Differentially expressed genes (DEGs) were selected by DESeq2 with a false discovery rate (FDR) below 0.05, and absolute fold change ≥ 2. Other analyses, such as GO, KEGG, and PCA, were performed using the OmicShare tools (https://www.omicshare.com/tools). The RT-qPCR process was same as the previous research [22]. For each gene, four biological replicates were used in RT-qPCR experiments.

### Gene cloning and enzyme expression

Proteins of the (+)-pulegone reductase (*MpPR*) gene from *Mentha piperita* and the (−)-pulegone reductase gene (*NtPR*) from *Nepeta tenuifolia* were used as query sequences [31] and the RNA-Seq were used as subject sequences. The E-value was set as 1 × 10^− 5^, and the sequences with the lowest “Weight Coverage” were homologous using TBtools [32]. The coding sequences (CDs) of candidate genes were PCR amplified using specific primers (Table S6). The CDs were cloned into the pET28a vector using Gibson assembly, and each plasmid was sequenced to verify sequence accuracy. The recombinant plasmid was then transformed into *Escherichia coli* strain *BL21* (DE3) (Invitrogen). Transformation was verified by colony PCR, and a positive colony was used to inoculate LB medium (5 mL) containing 100 ng L^− 1^ kanamycin. The culture (1 mL) was transferred into 100 mL of LB and incubated with agitation at 37℃ to reach an OD_600_ of approximately 0.8-1.0. The culture was induced by isopropyl β-thiogalactopyranoside (IPTG) to a final concentration of 1.0 mM, before being incubated at 37℃ for 16 h. Cells were harvested by centrifugation at 5000 *g* at 4℃ for 15 min and resuspended in buffer A (10 mM Tris-HCl, pH 8.0, 200 mM NaCl, and 5% glycerol). The suspension was transferred to an ultrasonic cell disruptor system and ultrasonically disrupted for 25 s each time, with 35 s on ice between disruptions. The mixture was centrifuged (30 min, 4℃, 12,000 *g*) and the supernatant was carefully collected as expressed protein. The expressed protein molecular weight was determined by SDS-PAGE using a protein marker (BeyoColor™ Prestained Color Protein Marker from Beyotime Biotechnology Co. LTD, Shanghai, China). The MpPR and NtPR protein were obtained as previous reported [31].

### Leaf protein extraction

The collected leaves were frozen in liquid nitrogen and ground into a powder using a mortar and pestle. Ice-cold extraction buffer (100 mM Tris-HCl, pH 8.0, 100 mM EDTA, 1 mM PMSF, 100 mM KCL, 10 mM Na_2_SO_3_, and 100 mM glycine) was used to extract total proteins [33]. A total of 1.5 g powder was added to a 10 mL centrifuge tube, 800 µL extraction buffer was added, and the mixture was vortexed for 20 min on ice and mix once per 5 min vortex. The mixture was centrifuged at 12,000 g for 15 min and the supernatant was quantified using the bicinchoninic acid (BCA) method.

### Protein enzymatic assays

The enzymatic reactions of extracted total proteins from leaves were performed as follows: 100 µL total proteins, 230 µL buffer (KH_2_PO_4_ 50 mmol L^− 1^, 10% sorbitol, Dithiothreitol (DTT) 1 mmol L^− 1^, pH 7.5), 10 mM NADPH (40 µL), 10 mM G6P (20 µL), 10 mM G6PDH (10 µL), and 10 mM substrate (0.8 µL), and then 0.2 mL of n-hexane was added to the top of the reaction solution [31]. Reactions were carried out at 31℃ for 16 h with slow stirring and terminated by placing the reaction vial at -80℃ for 1 h. The top organic phase of the mixture was analyzed by GC-MS. A negative control was performed by the same reaction, and the protein was heated for 15 min at 95℃. The enzymatic reactions of expressed proteins were performed by including 280 µL buffer, 10 mM NADPH (40 µL), 10 mM G6P (20 µL), 10 mM G6PDH (10 µL), expressed protein (50 µL), and 10 mM substrate (5 µL), which reactions were used isoegoma ketone, pulegone, 3-nonen-2-one as substrates. Then 0.2 mL of n-hexane was added to the top of the reaction solution. Reactions were carried out at 31℃ for 16 h with slow stirring and terminated by placing the reaction vial at -80℃ for 1 h. The top organic phase of the mixture was analyzed by GC-MS. The GC-MS protocol was the same as that used for volatile oil analysis.

The reactions of enzyme activity with 4-hydroxybenzalacetone, artemisinic acid, and artemisitene as substrates and incubation condition were same as above. The reactions was stopped by adding 250 µL of methanol. Next, all samples were centrifuged at 12,000 g for 10 min and, the supernatant was subjected to HPLC analysis. A Waters Acquity HPLC™ system (e2695; Waters Corp., MA, USA) equipped with a TUV detector system (2998; Waters Corp.) was used to analyze enzyme activity. Chromatography was performed on an XBridge C18 column (250 m × 4.6 mm, 5 μm). The mobile phase consisted of acetonitrile (A) and 0.2% formic acid in water (B). The HPLC elution conditions were optimized as follows: 0–10 min, 10-20% A; 10–28 min, 20–30% A; 28–33 min, 30 − 10% A; 33–37 min, 10%. The flow rate was 1.0 mL/min, the column was maintained at 30℃, and the injection volume was set as 10 µL.

## Electronic supplementary material

Below is the link to the electronic supplementary material.

Supplementary Material 1

Supplementary Material 2

## Data Availability

The datasets generated and/or analyzed during the current study are available in the Sequence Read Archive (SRA repository), under the Accession number PRJNA880738 [https://www.ncbi.nlm.nih.gov/bioproject/PRJNA880738] at NCBI.

## Abbreviations

C: Transcitral
DBR: Double bond reductase
DMAPP: Dimethylallyl diphosphate
EK: Elsholztiaketone
GeDH: Geraniol dehydrogenase
GES: Geraniol synthase
GPP: Geranyl diphosphate
GPPS: Geranyl diphosphate synthase
IPP: Isopentenyl diphosphate
DMAPP: Dimethylallyl diphosphate
MDR: Medium-chain dehydrogenase/reductase
MEP: 2-C-methyl-D-erythritol-4-phosphate pathway
PA: Perillaldehyde
PK: Perilla ketone
PL: Perillene
PP: Phenylpropanoid
PT: Piperitenone
TCM: Traditional Chinese medicine
RT-qPCR: Real-time quantitative PCR
GT: Glandular trichomes
PGT: Peltate glandular trichome
DXS: 1-deoxy-D-xylulose-5-phosphate synthase
DXR: 1-deoxy-D-xylulose-5-phosphate reductoisomerase
MCS: 2,4-cyclodiphosphate synthase
HDS: (E)-4-hydroxy-3-methylbut-2-enyl-diphosphate synthase
HDR: 4-hydroxy-3-methylbut-2-en-1-yl diphosphate reductase
MCT: 2-C-Methyl-D-eryth-ritol 4-phosphate cytidylyltransferase
CMK: 4-diphosphocytidyl-2-C-methyl-D-erythritol kinase
DOXP: 1-Deoxy-D-xylulose 5-phosphate
MEP: 2-C-Methyl-D-erythritol 4-phosphate
CDP-ME: 4-(Cytidine 5’-diphospho)-2-C-methyl-D-erythritol
CDP-MEP: 2-Phospho-4-(cytidine 5’-diphospho)-2-C-methyl-D-erythritol
MECP: 2-C-Methyl-D-erythritol 2,4-cyclodiphosphate
HMBPP: 1-Hydroxy-2-methyl-2-butenyl 4-diphosphate
AACT: Acetyl-CoA acetyltransferase
HMGS: Hydroxymethylglutaryl-CoA
HMGCR: Hydroxymethylglutaryl-CoA reductase synthase
MVK: Mevalonate kinase
PMK: Phosphomevalonate kinase
MVD: Mevalonate diphosphate decarboxylase
MVA: Mevalonic acid
MVA-5-P: Mevalonic acid 5-phosphate
MVAPP: 5-Diphosphomevalonic acid
FPP: Farnesyl diphosphate
