# Supplementary material for: The revealing of a novel double bond reductase related to perilla ketone biosynthesis in Perilla frutescens
Source: BMC Plant Biol. 2023 Jun 30;23:345. doi: 10.1186/s12870-023-04345-1 (PMC10311769; doi:10.1186/s12870-023-04345-1)
Supplement: Supplementary file 2 — Supplementary Material 2 [file 12870_2023_4345_MOESM2_ESM.pdf]

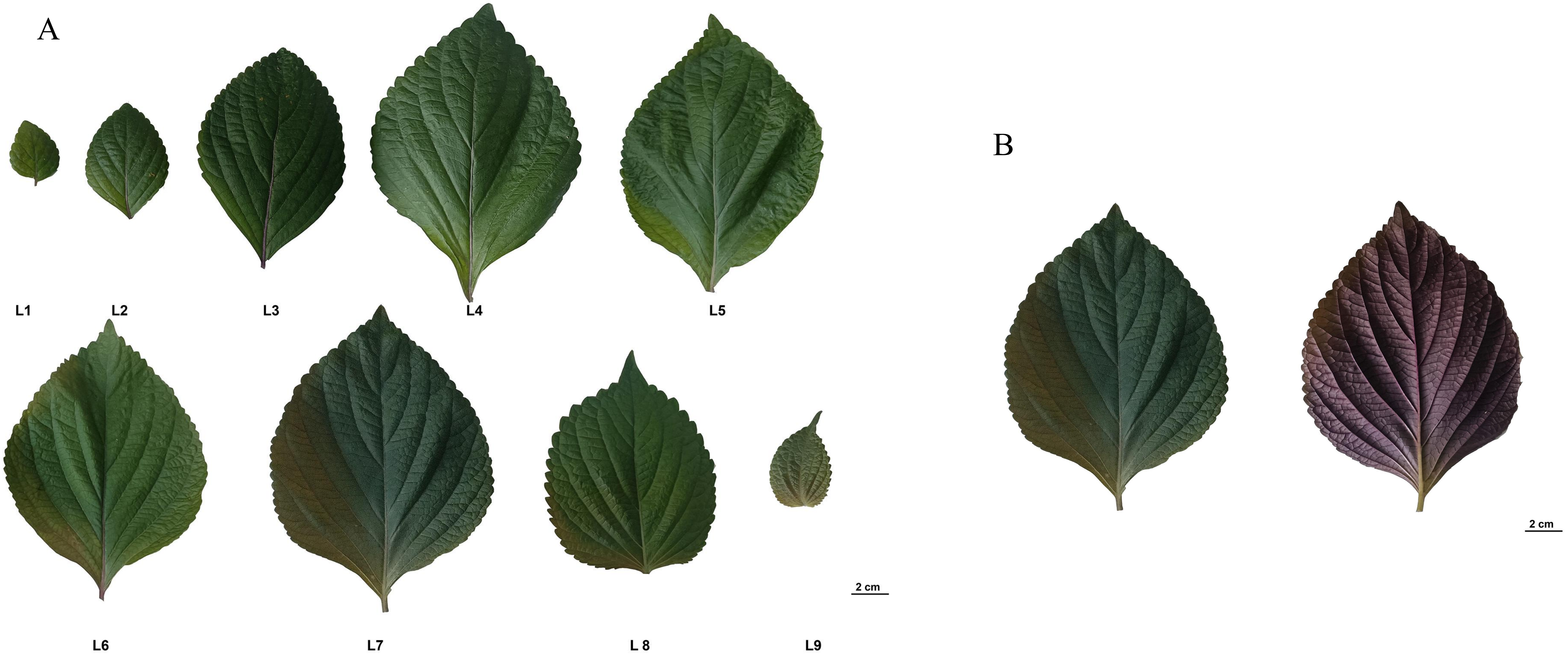

Fig.S1 The morphology of different levels of leaves. (A) The plant materials of different level leaves; (B) The color of paraxial and abaxial leaves of *P. frutescens*.

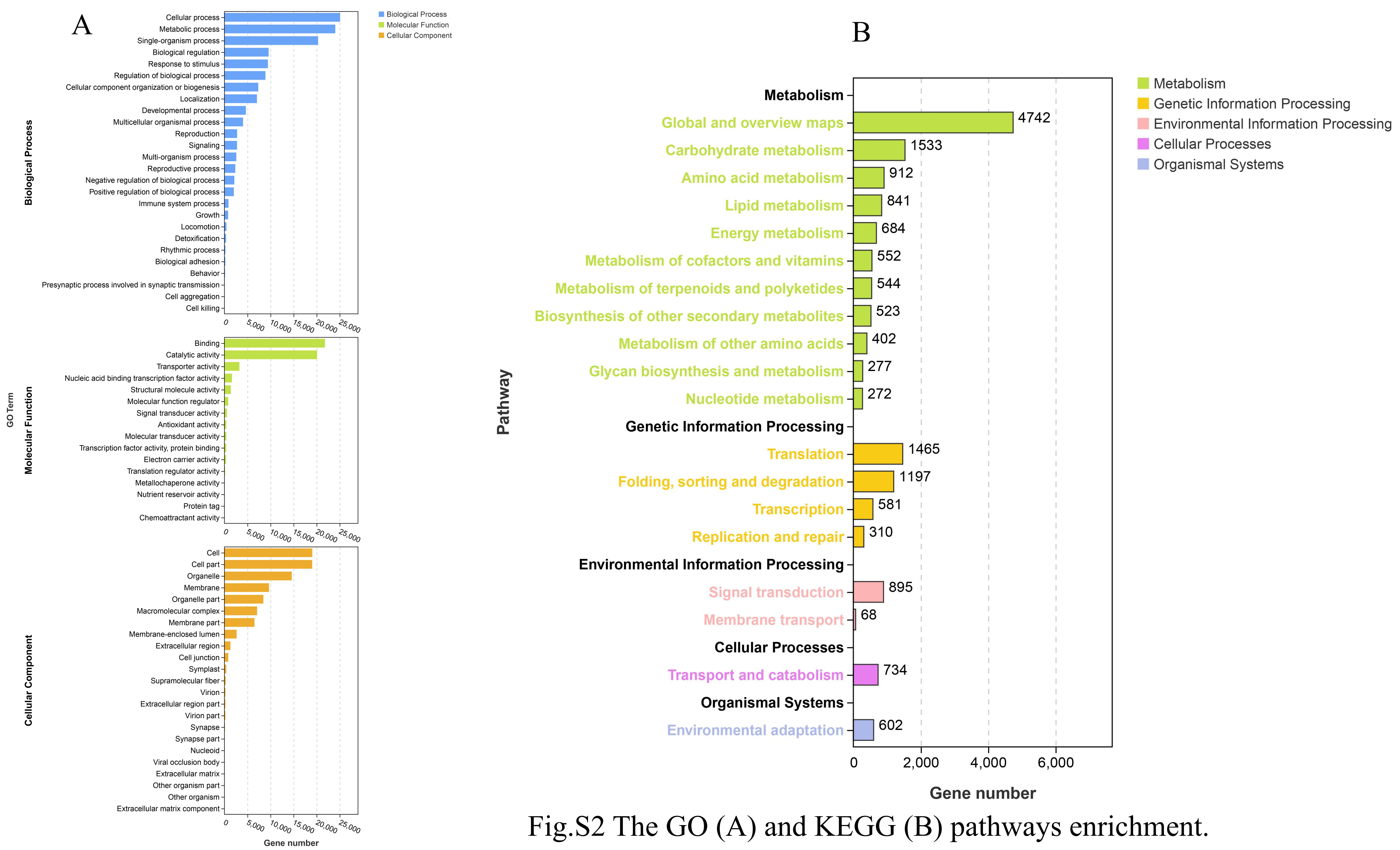

Fig.S2 The GO (A) and KEGG (B) pathways enrichment.

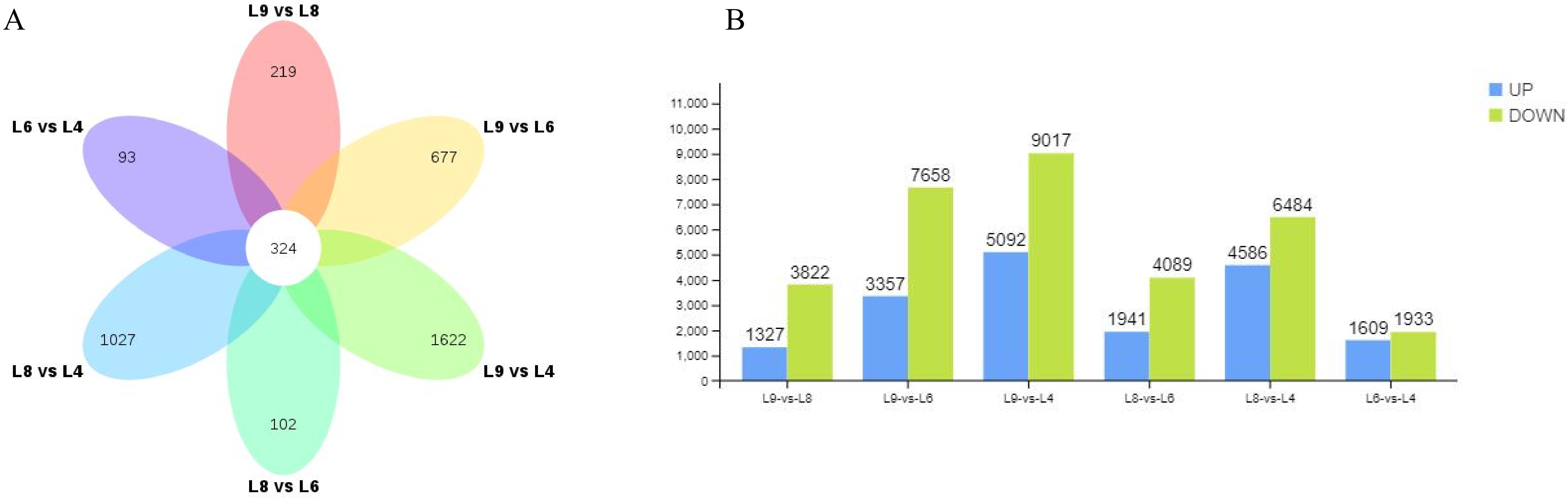

Fig.S3 The different expressed genes among six comparison groups. (A) The venn of DEGs. (B) The number of DEGs in different groups.

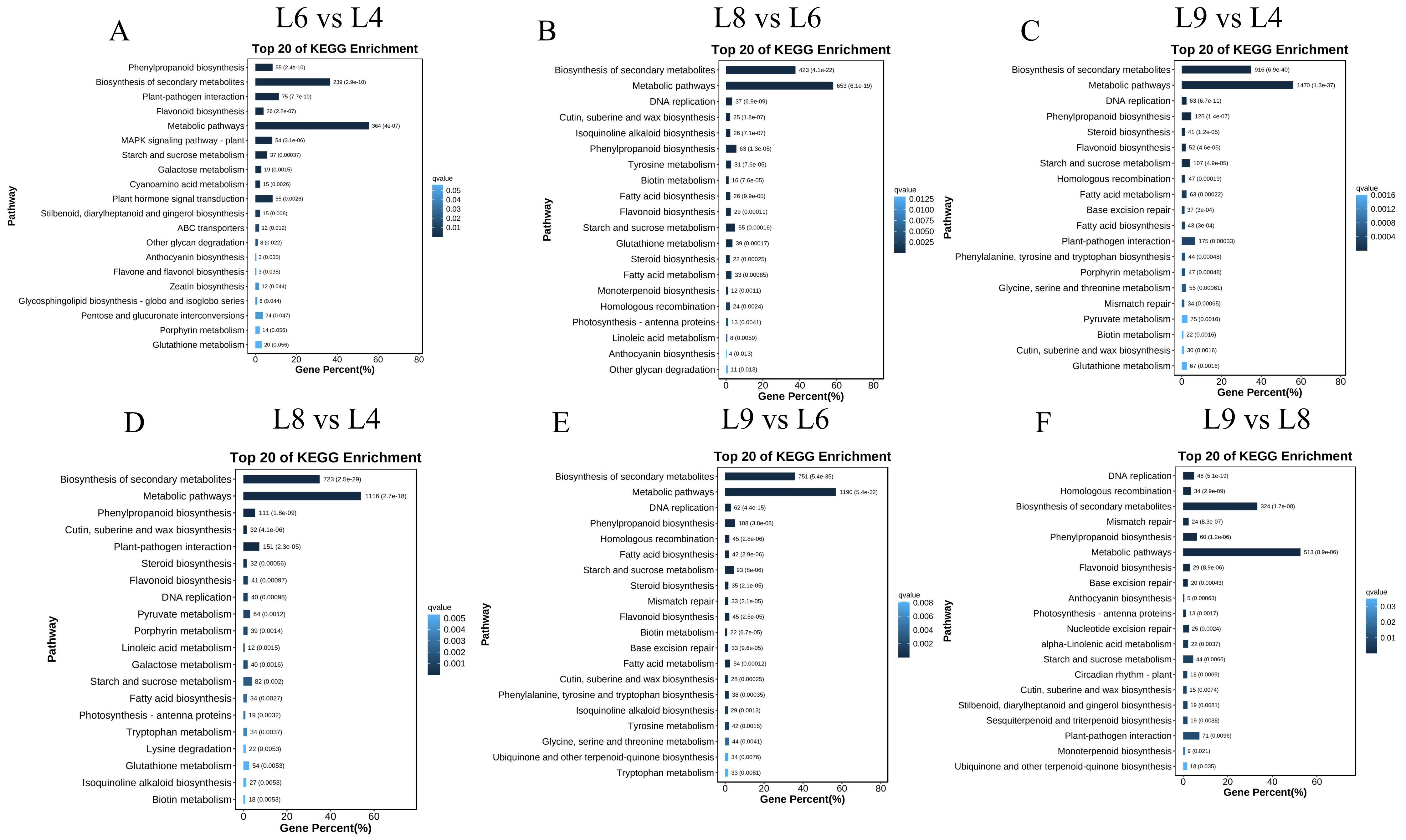

Fig.S4 The top 20 KEGG enrichment pathways of six comparison group.

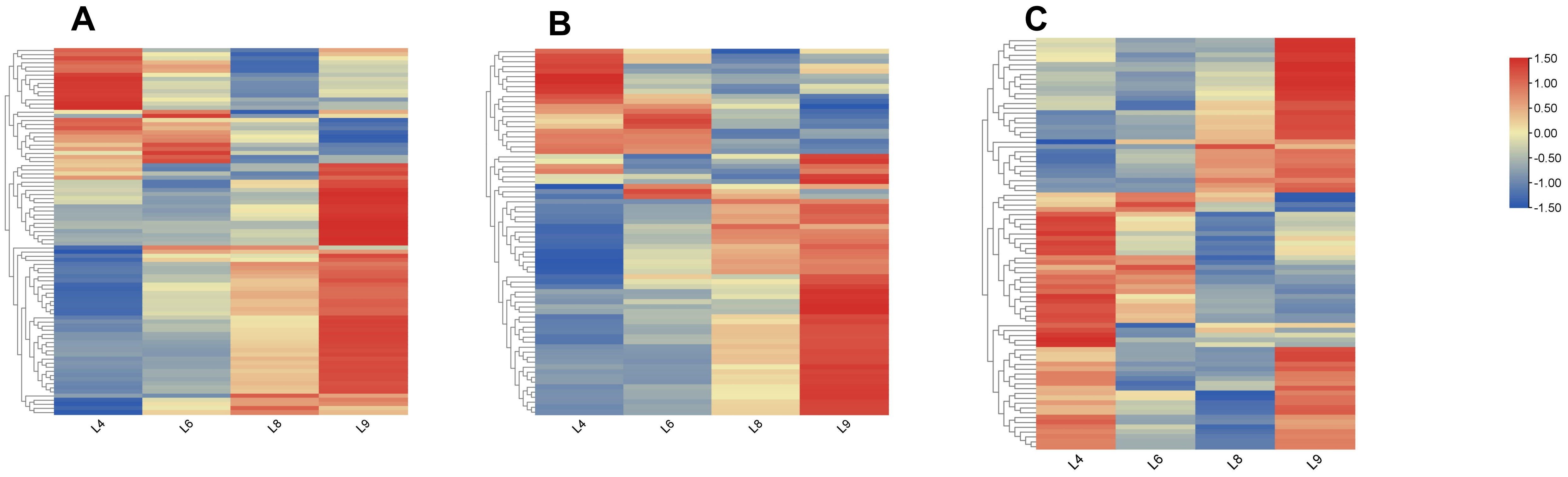

Fig. S5 The genes expression of MYB (A), bHLH (B), and AP2-ERF (C).

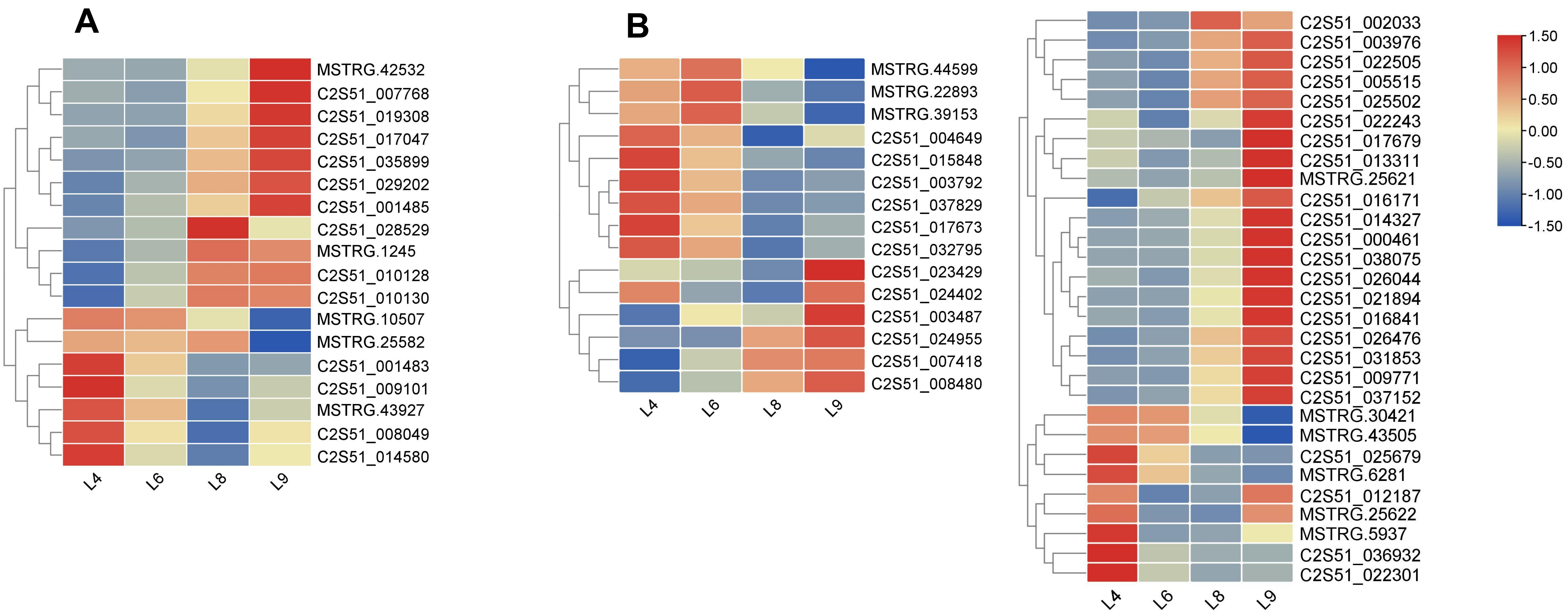

Fig. S6 The heatmap of genes expression involved in monoterpenoid biosynthesis (A), diterpenoid biosynthesis (B), and sesquiterpenoid and triterpenoid biosynthesis (C).

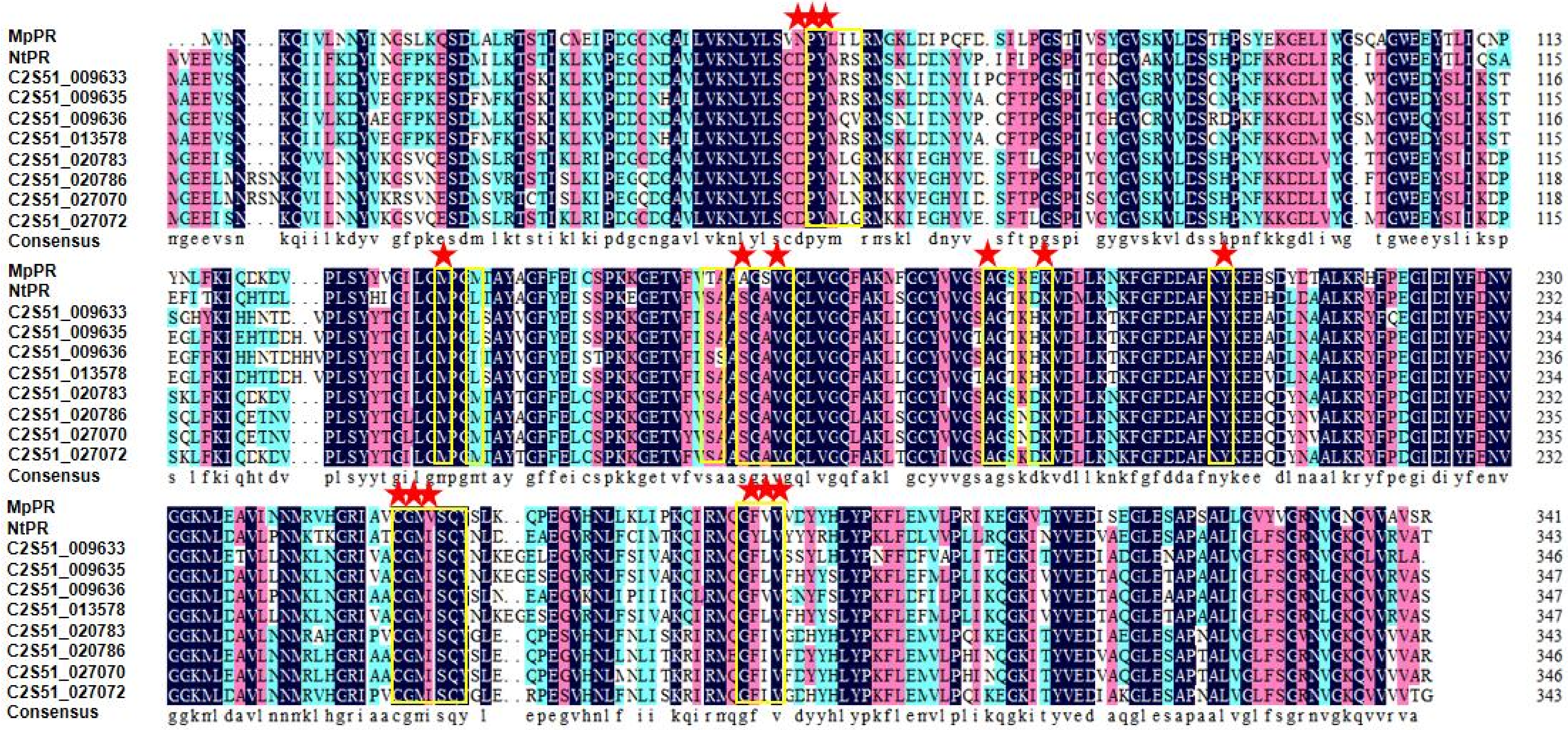

Fig.S7 Alignment of the DBRs polypeptide sequences of *P. frutescens* with those of NtPR and MpPR. The predicted residues in the NADP(H) binding site of are indicated with red five-pointed stars. Conserved residues involved in NADP(H) binding of those genes are indicated with yellow rectangle.

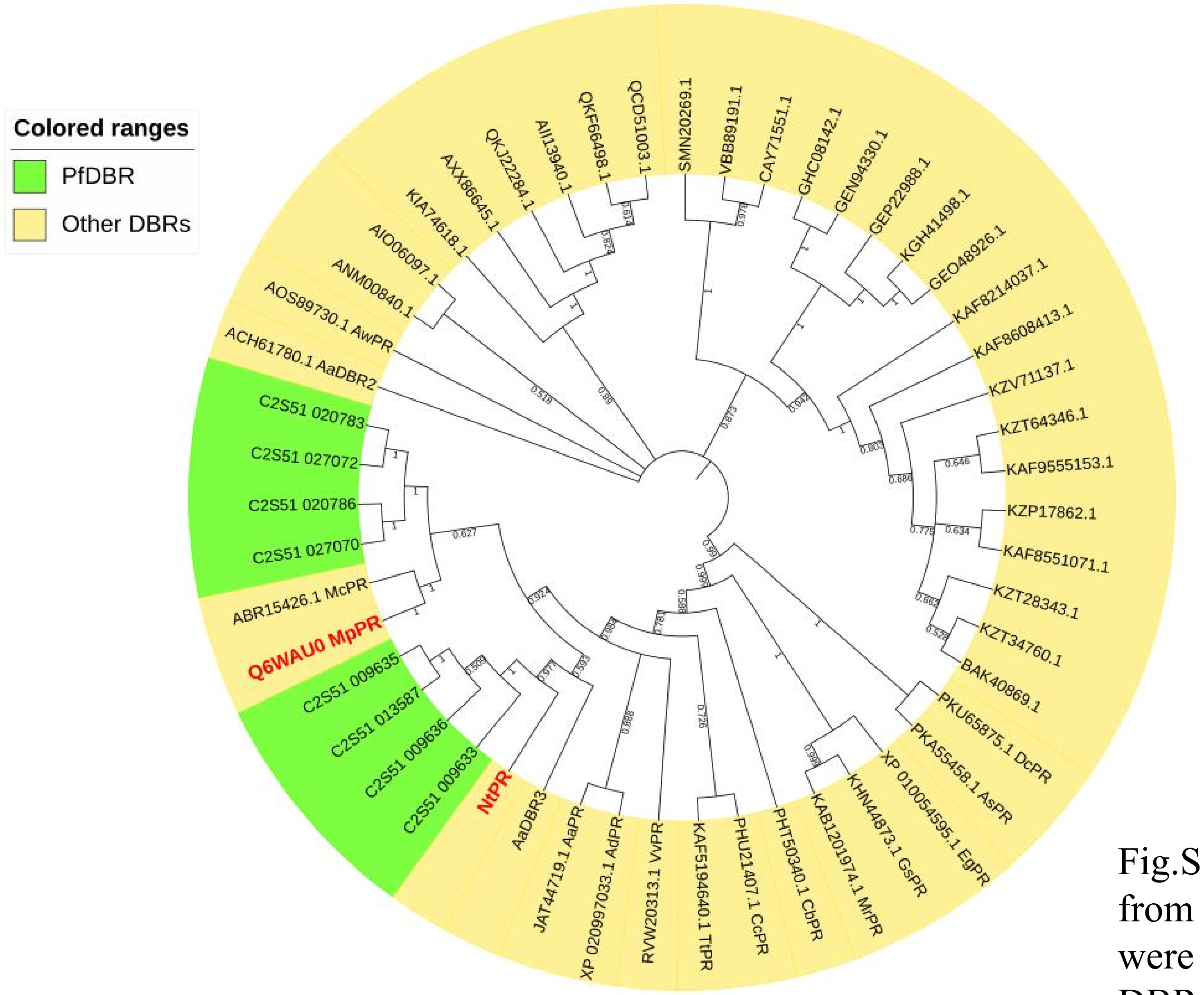

Fig.S8 Phylogenetic analysis of alkene reductases from the MDR superfamily. The DBRs of P. frutescens were highlighted in green background, and other DBRs were highlighted in yellow background. The tagret genes NtPR and MpPR were marked in red.

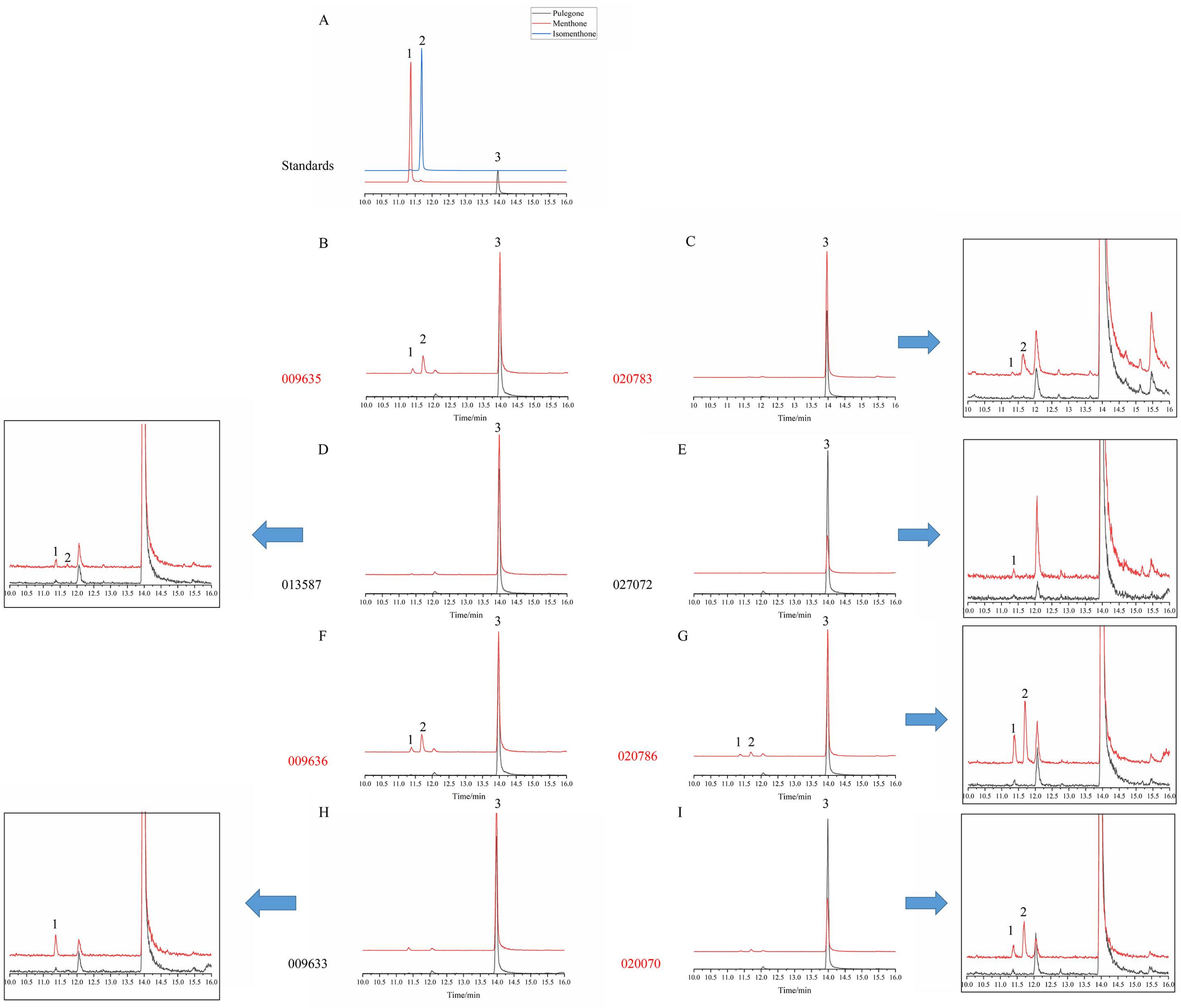

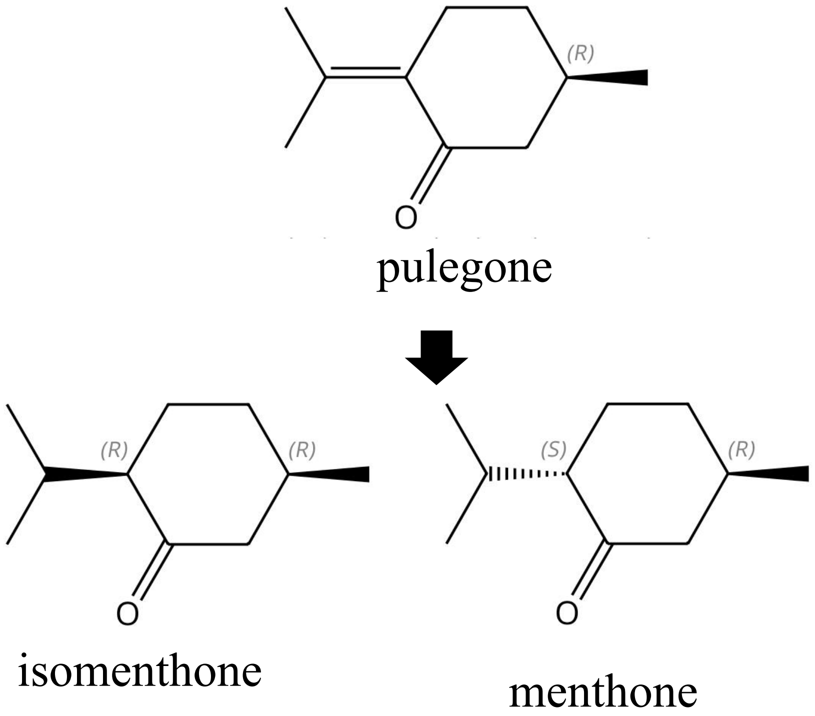

Fig.S9 The enzyme assays in vitro of eight proteins with the reaction of pulegone to menthone or isomenthone. Peak1, menthone wit RT 11.37 min; Peak 2, isomenthone with RT 11.63 min; Peak 3, pulegone with RT 14.01 min. The red line indicated the experimental group, and the black line indicated negative control. The protein in negative control was boiled and other ingredients were same as experimental group.

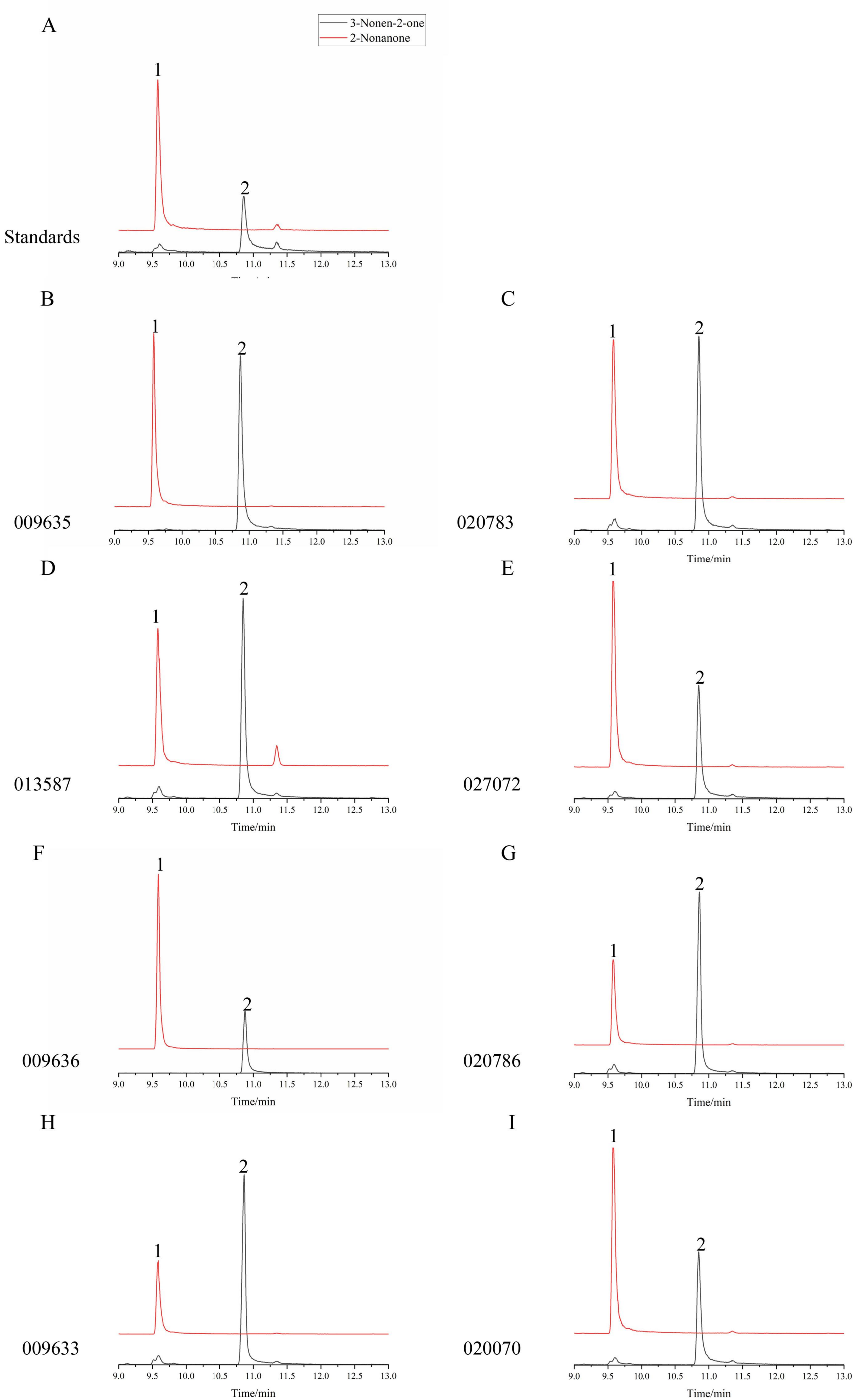

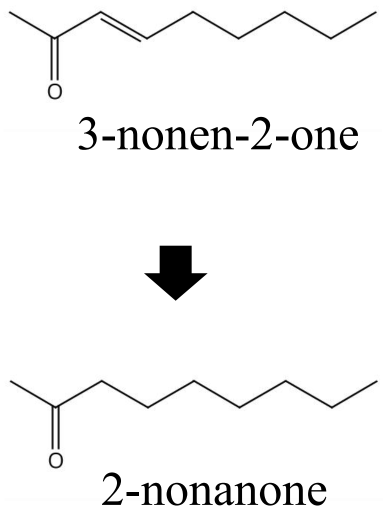

Fig.S10 The enzyme assays in vitro of eight proteins with the reaction of 3-nonen-2-one to 2-nonanone. Peak 1, 3-nonen-2-one with RT 10.83 min; Peak 2, 2-nonanone with RT 9.60 min. The red line indicated the experimental group, and the black line indicated negative control. The protein in negative control was boiled and other ingredients were same as experimental group.

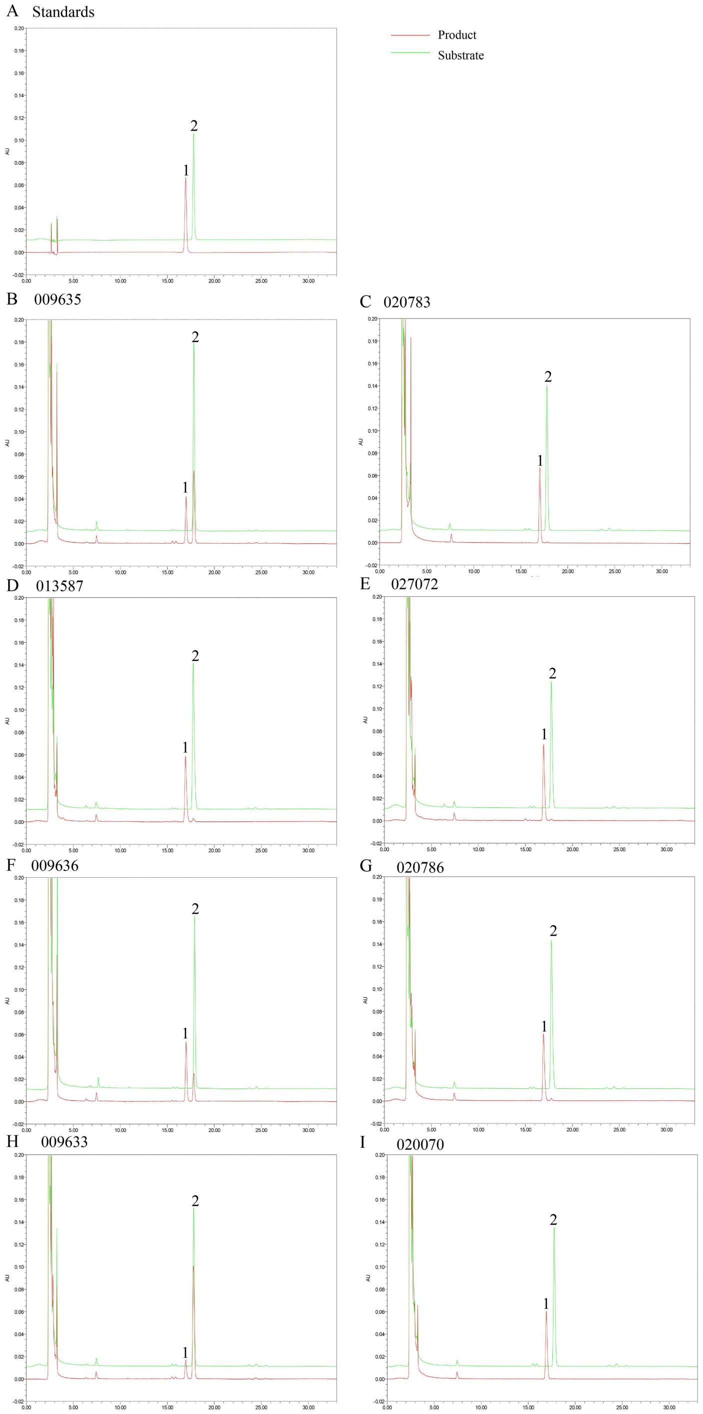

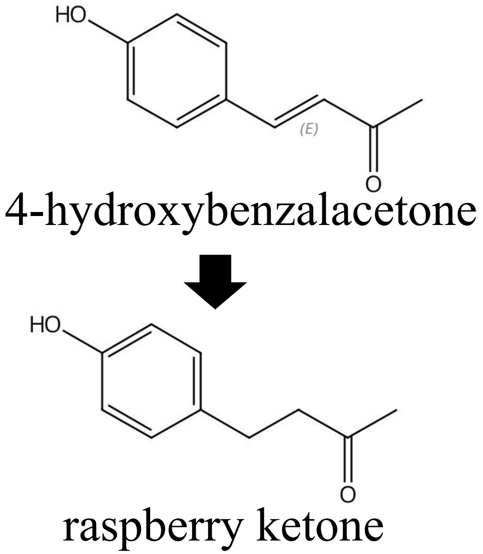

Fig.S11 The enzyme assays in vitro of eight proteins with the reaction of 4-hydroxybenzalacetone to raspberry ketone. Peak 1, raspberry ketone with RT 16.90 min; Peak 2, 4-hydroxybenzalacetone with RT 17.78 min. The green line was the control group and the red line was the experimental group. The protein in negative control was boiled and other ingredients were same as experimental group.

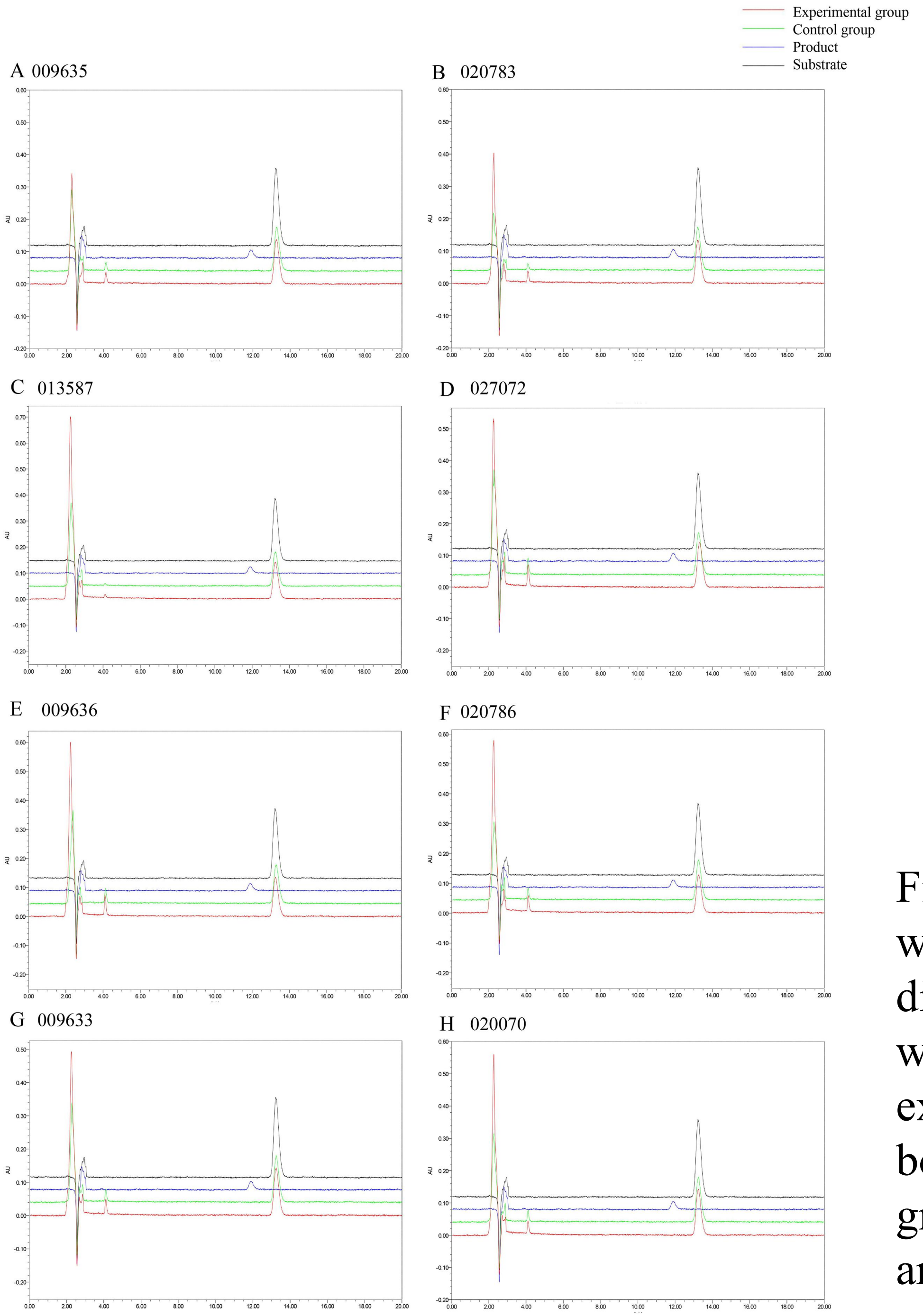

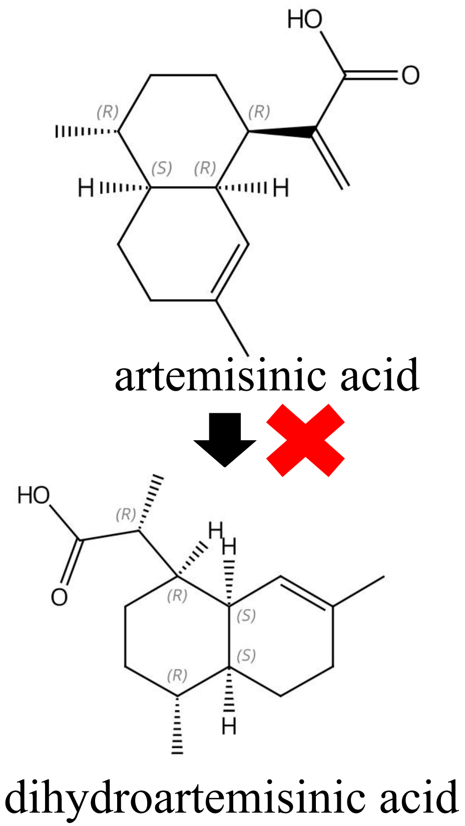

Fig.S12 The enzyme assays in vitro of eight proteins with the reaction of artemisinic acid (RT 13.25 min) to dihydroartemisinic acid (RT 11.71 min). The green line was the control group and the red line was the experimental group. The protein in negative control was boiled and other ingredients were same as experimental group. The black and blue line was the standards of artemisinic acid and dihydroartemisinic acid, respectively.

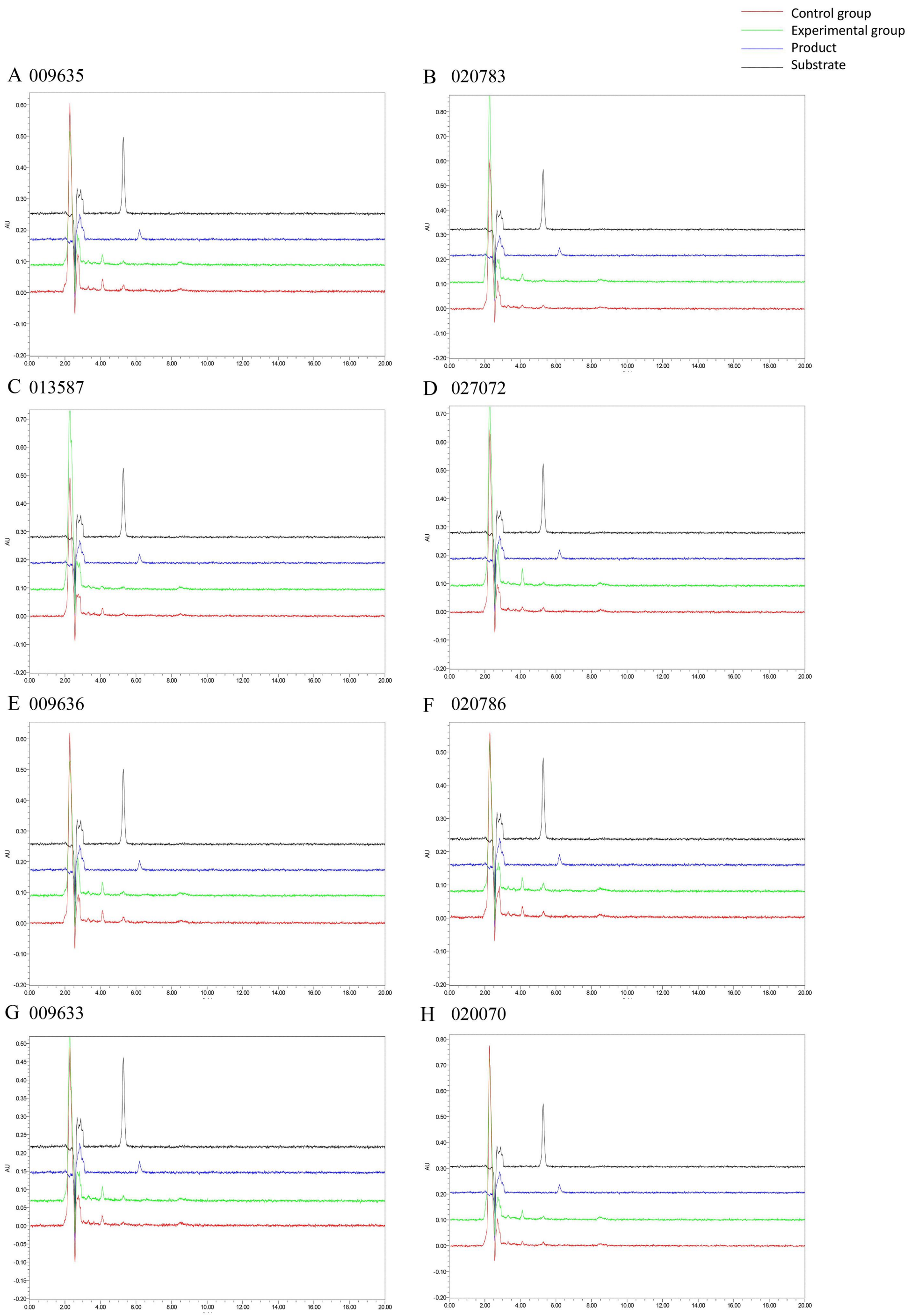

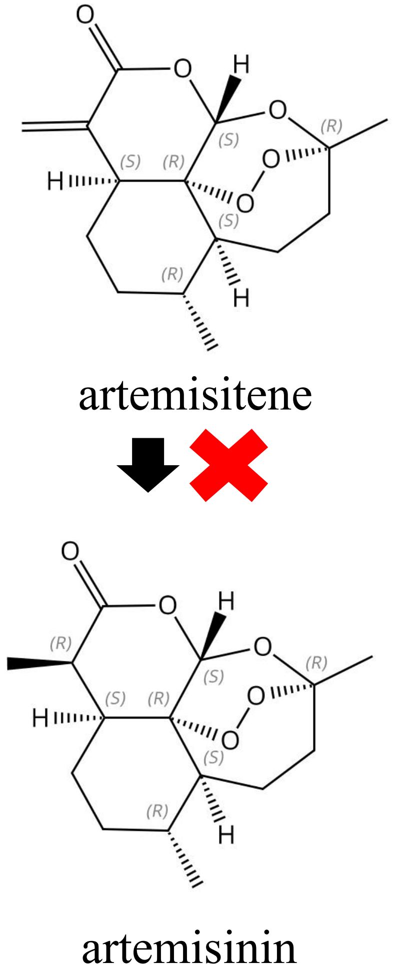

Fig.S13 The enzyme assays in vitro of eight proteins with the reaction of artemisitene (RT 5.38 min) to artemisinin (RT 6.25 min). The red line was the control group and the green line was the experimental group. The protein in negative control was boiled and other ingredients were same as experimental group. The black and blue line was the standards of artemisinic acid and dihydroartemisinic acid, respectively.

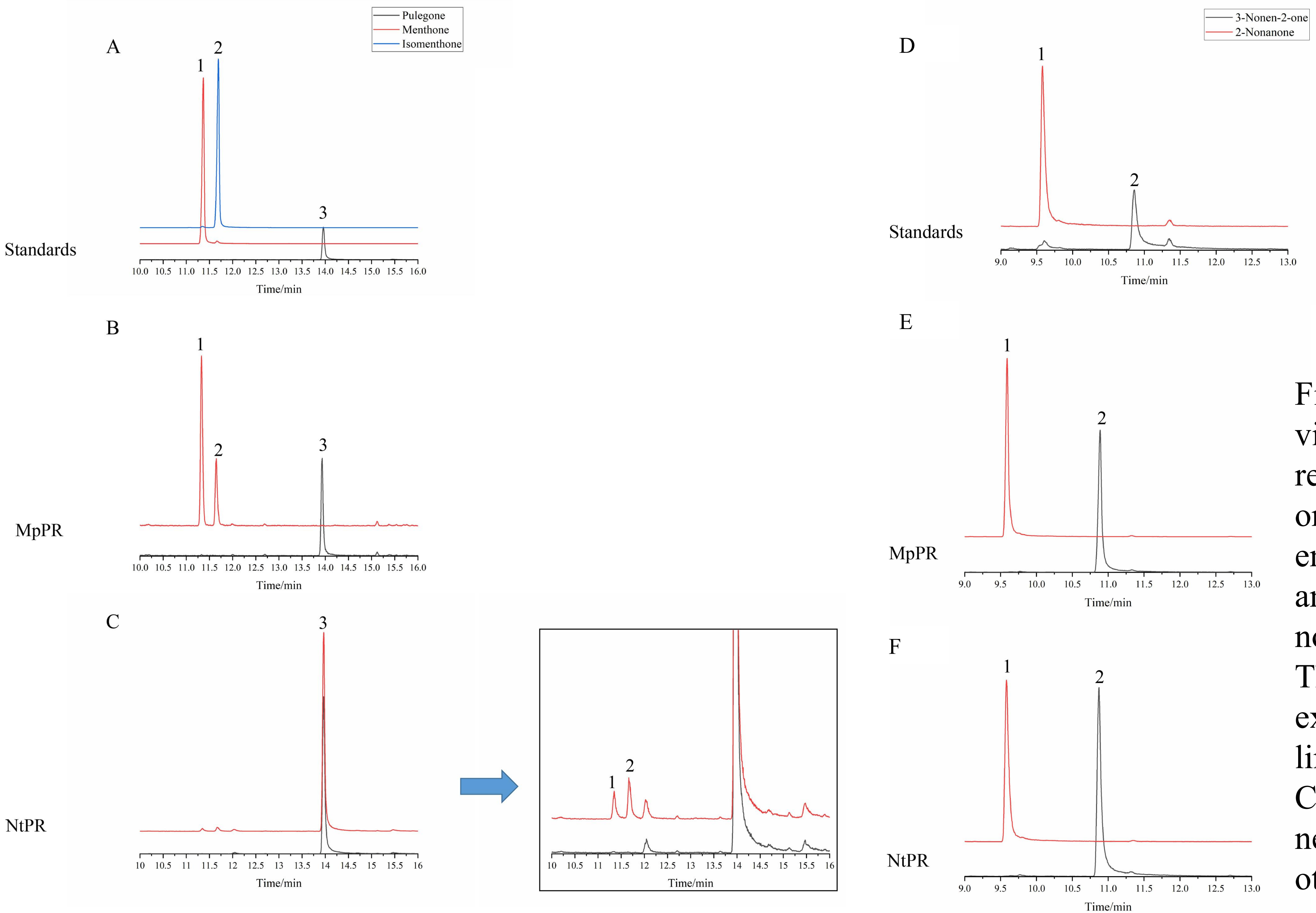

Fig.S14 The enzyme assays in vitro of NtPR and MpPR with the reaction of pulegone to menthone or isomenthone (A-B); The enzyme assays in vitro of NtPR and MpPR with the reaction of 3-nonen-2-one to 2-nonanone (D-F). The red line indicated the experimental group, and the black line indicated negative control in B, C, E, and F. The protein in negative control was boiled and other ingredients were same as experimental group.

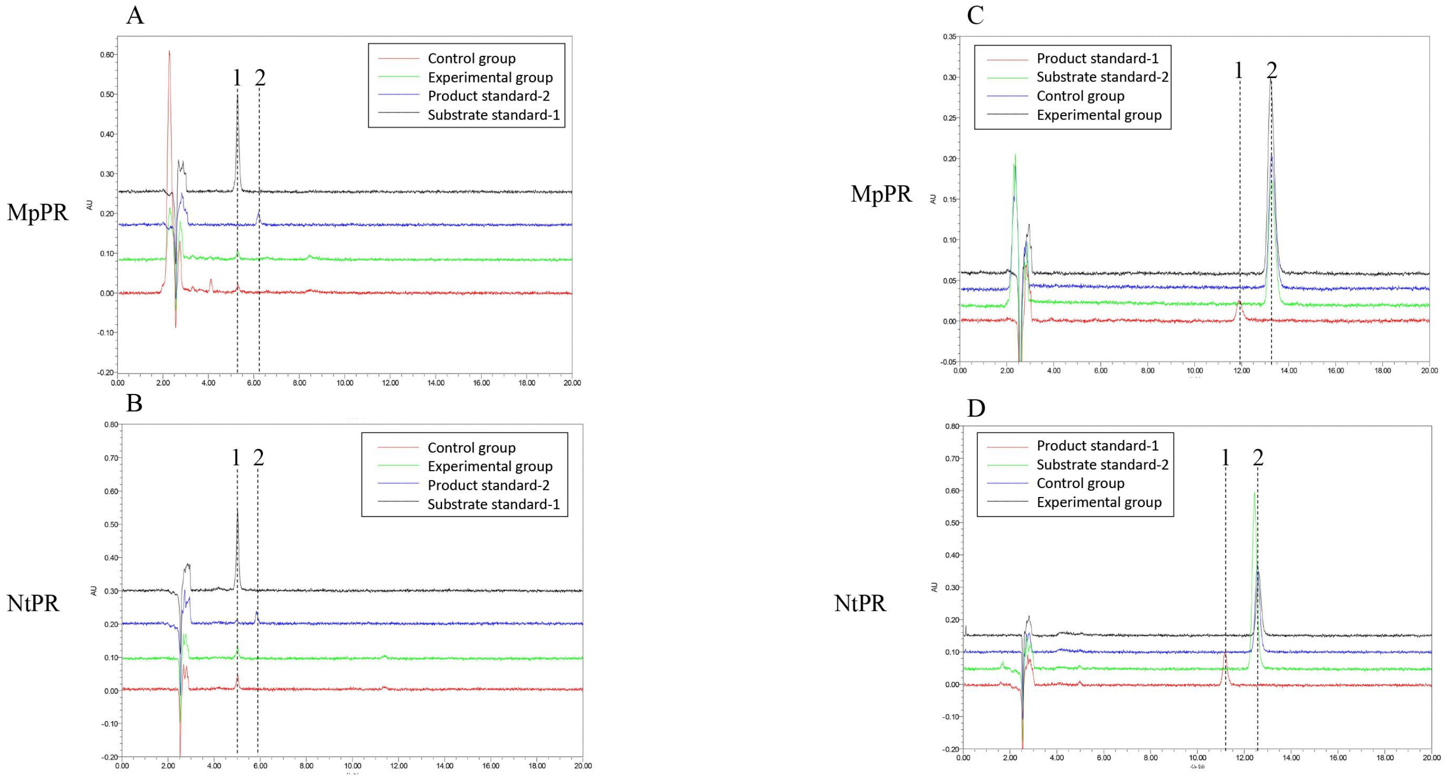

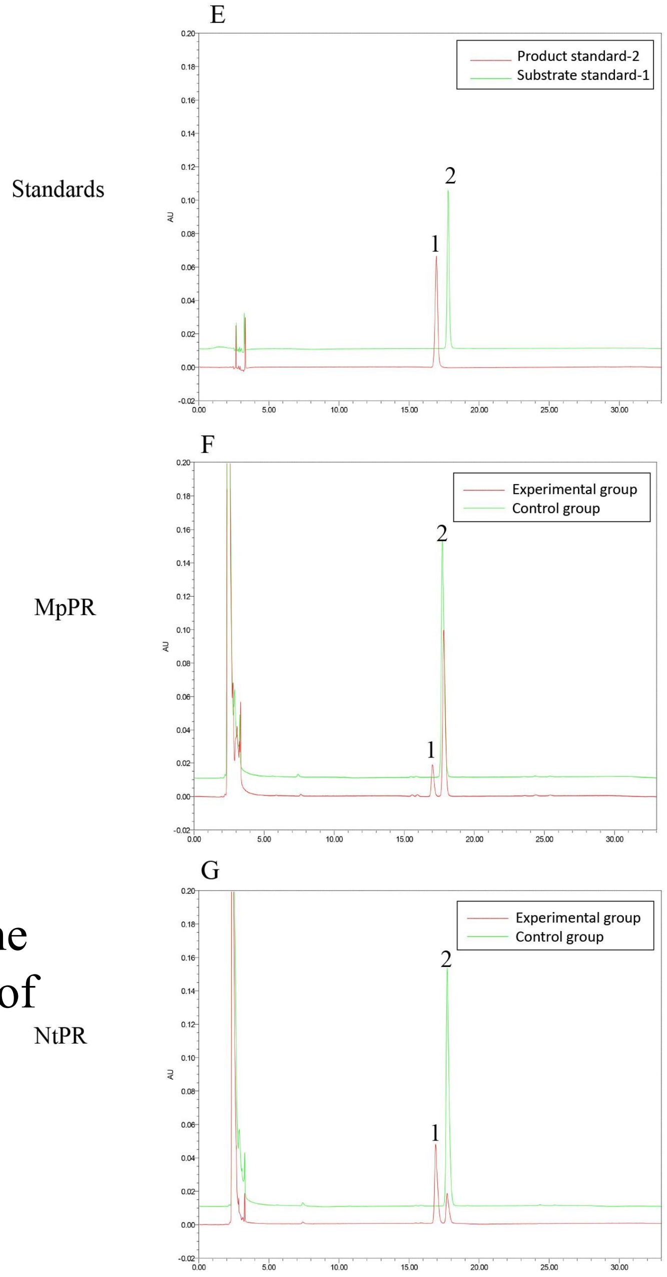

Fig.S15 The enzyme assays in vitro of NtPR and MpPR with the reaction of artemisitene to artemisinin (A-B); The enzyme assays in vitro of NtPR and MpPR with the reaction of artemisinic acid to dihydroartemisinic acid (C-D). The enzyme assays in vitro of NtPR and MpPR with the reaction of 4-hydroxybenzalacetone to raspberry ketone (E-F). The protein in negative control was boiled and other ingredients were same as experimental group.

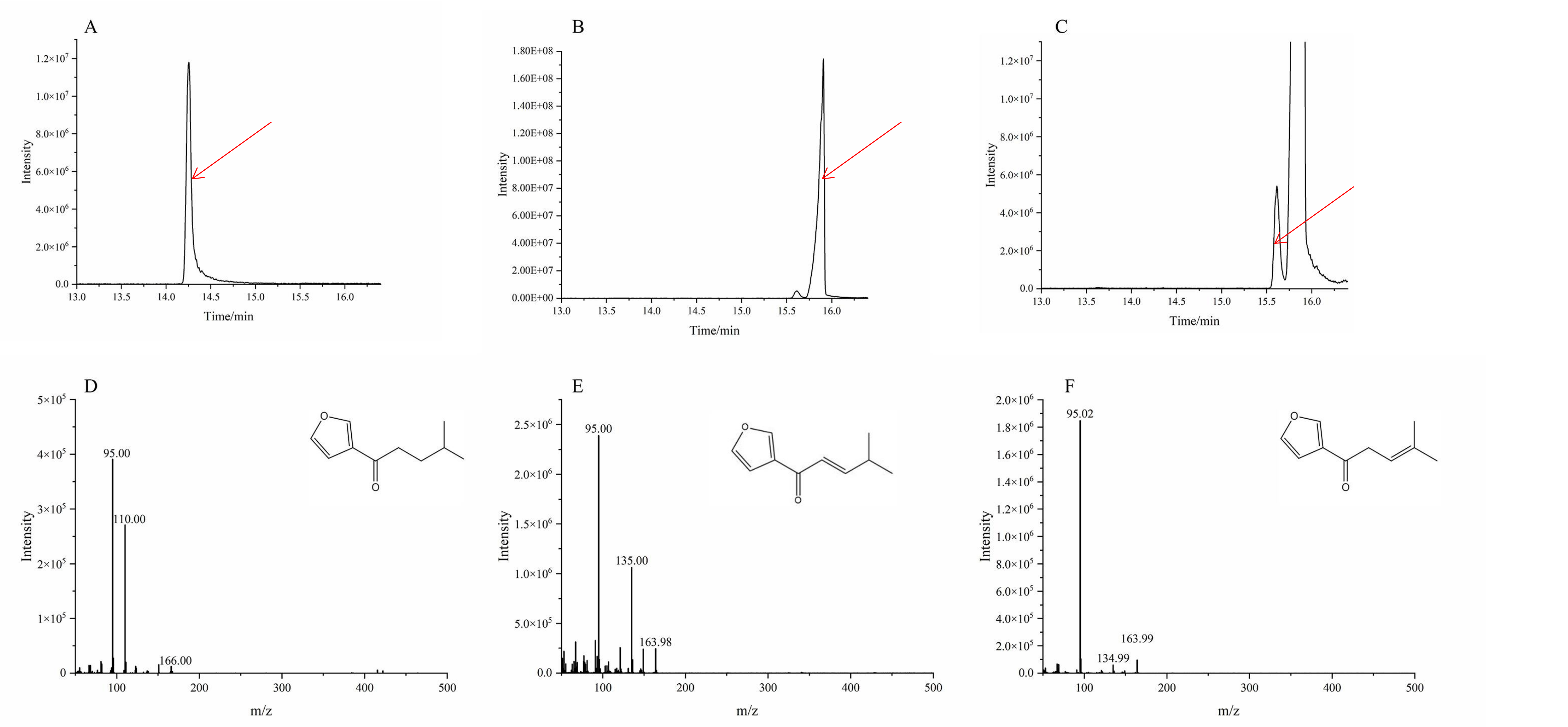

Fig.S16 The GC-MS and MS of perillaketone (A, D, RT 14.15 min), isoegomaketone (B, E, RT 15.86 min) and egomaketone (C, F, RT 15.61 min) standards.

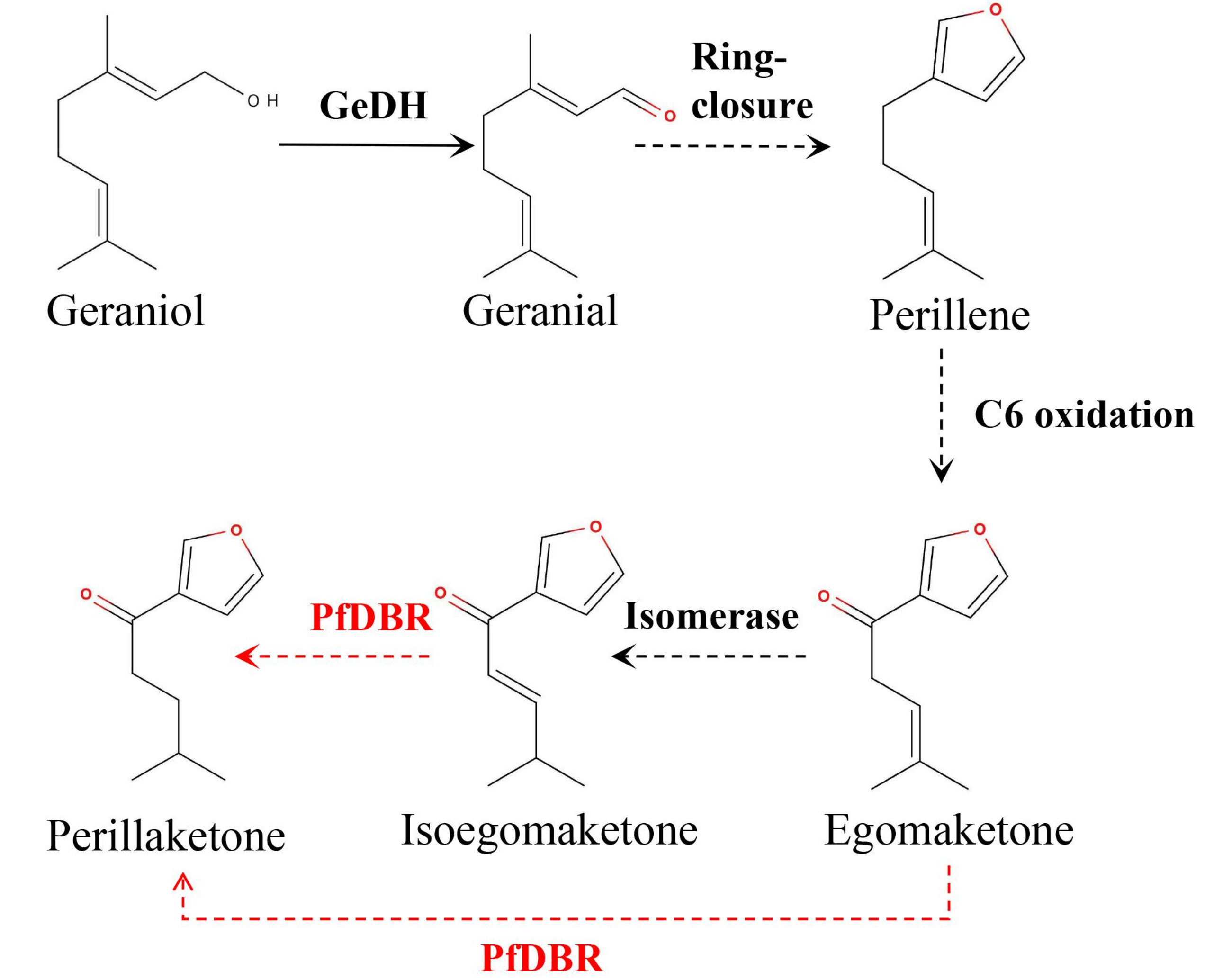

Fig.S17 The proposed PK biosynthesis.
